# Status and trends of RGS16 based on data visualization analysis: A review

**DOI:** 10.1097/MD.0000000000036981

**Published:** 2024-02-16

**Authors:** Liu Wenbo, Xie Liangyu, Lu Zhiyong, Yu Gongchang, Chen Yuanzhen, Shi Bin

**Affiliations:** a Bone Biomechanics Engineering Laboratory of Shandong Province, Neck-Shoulder and Lumbocrural Pain Hospital of Shandong First Medical University, Shandong First Medical University and Shandong Academy of Medical Sciences, Jinan, China; b Shandong Traditional Chinese Medicine University, Jinan, Shandong Province, China.

**Keywords:** allergic and irritant contact dermatitis, cancer, RGS16, schizophrenia, visualization study

## Abstract

G-protein signaling regulator 16 (RGS16) has been confirmed that RGS16 is associated with cancer, neurodegenerative diseases, and cardiovascular diseases. Moreover, many studies have shown that RGS16 can be used as a biomarker for cancer diagnosis and prognosis. We used CiteSpace and VOS viewer software to perform a bibliometric analysis of 290 publications in the core collection of Web of Science. All the articles come from 399 institutions, including 618 authors, 179 journals, 40 countries, 115 keywords, 1 language, two types of papers, and reviews. The United States has the largest number of publications. The Research Center of Allergy and Infectious Diseases (NIAID) publishes the most papers, Emory University is the most recent of all institutions with the most recent results in the RGS16 study. Cell biology is the most studied discipline, and the most studied topic is migration. Drury published RGS16-related articles with the most citations (n = 15), and Berman published articles with the most citations (n = 106). The biological applications of RGS16 are currently a hot area of RGS16 research, including inflammation, cancer, ulcerative colitis, metabolic acidosis, platelet activation, and thrombosis. The current scientometrics study provides an overview of RGS16 research from 1995 to 2022. This study provides an overview of current and potential future research hotspots in the field of RGS16 and can be used as a resource for interested researchers.

## 1. Introduction

G-protein signaling regulator 16 (RGS16) belongs to the RGS protein B/R4 subfamily with RGS1-5, 8, 13, 18, and 21 due to their similar molecular size (~20–25 kDa).^[1]^ It has been confirmed that RGS16 is associated with cancer, neurodegenerative diseases, and cardiovascular diseases.^[2]^ It can also act through multiple signaling pathways. The activity and function of RGS16 protein can be influenced by phosphorylation and palmitoylation.^[3,4]^ Moreover, many studies have shown that RGS16 can be used as a biomarker for cancer diagnosis and prognosis, and scholars have gradually paid more and more attention in recent years.^[5]^

This study used CiteSpace software, VOS viewer,^[6]^ and Excel, to visually and systematically describe the trajectory and current status of RGS16 research, and to address the research frontiers and emerging trends in the RGS16 literature. The present results will provide a framework for relevant researchers.^[7]^ We used the keyword screening time for the earliest RGS16 literature^[8]^ to the start of the study, i.e., April 1995 to December 2022. We ended up with 322 literature articles. After duplicate removal, a total of 290 usable literature articles were obtained from 40 countries/regions, 399 institutions, 618 authors, 179 journals with a total of 115 keywords, and 1 language, containing both treatise and review types of literature.

To our knowledge, there have been no bibliometrics studies on RGS16 to date. Therefore, this study aims to provide an in-depth analysis and visualization of RGS16-related research between 1995 and 2022 and to assess the current state and future trends of research in this field (mainly cancer, inflammation, and schizophrenia).

## 2. Data acquisition and methods

### 2.1. Data acquisition

Through a variety of techniques, including time slicing, thresholding, modeling, pruning, merging, and mapping, we follow the fundamental steps of data visualization.^[9]^ We have developed a clearer search strategy and screening method to get the literature data we need quickly.

The web of science core collection (WoSCC) was the article search resource used for the analysis, which is often used in data visualization studies.^[10]^ When we searched the core collection data across all fields using the 2 subject terms “RGS16” and “Regulator of G-protein signaling 16,” we were able to find the majority of articles that dealt with RGS16. The search results have been filtered by research area, article type, and language type.^[11]^ We renamed the file “download_*.txt” so that the data could be analyzed by CiteSpace.

**Figure 1.**
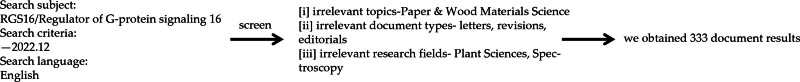
The flow chart of literature screening included in this study.

Using (topic = [“RGS16” or “regulator of G-protein signaling 16”]) and (language = [English]) as search formulae, we obtained 333 document results (Fig. 1). To improve the accuracy of the information, for this purpose, several categories need to be excluded, such as irrelevant topics like Paper & Wood Materials Science, irrelevant document types such as letters, revisions, editorials, and irrelevant research fields like Plant Sciences, Spectroscopy.^[12]^ After a relatively rigorous literature screening, we obtained relatively convincing data, although there may have been some literature excluded that should have been included, we have reason to believe that it is not closely related to the purpose of our study.^[12]^

The search yielded 290 publications from April 1995 to October 2022. We then exported the bibliographic records in plain text format, including titles, abstracts, keywords, and references, for subsequent visual analysis of the data.^[6,10]^ Links to the relevant searches are provided in the supplementary material.

### 2.2. Data visualization and analysis methods

In this study, the annual publishing of RGS16 papers was counted and graphed using Microsoft Excel. Web of Science provided the journals’ impact factors (IF), Journal Citation Reports (JCR) divisions, and academics’ H-indexes.^[13]^ CiteSpace^[9]^ is an information visualization software developed by Chaomei Chen, a professor of computer and information science at Drexel University. This software is currently mostly used to analyze research trends in a field and present them in the form of visual graphs. We used CiteSpace 6.1.R3 software (Drexel University, Philadelphia, PA) to analyze the country/region, institution, author, keyword, journal, and reference co-citation features of RGS16 publications. In addition, Researchers at Leiden University, Nees Jan van Eck and Ludo Waltman, designed VOSviewer,^[6]^ a bibliometric analysis tool for knowledge mapping. It can be used for reference co-citation analysis and keyword co-occurrence analysis. It can display research findings and offers special benefits in terms of clustering methods and map display. For the study of term co-occurrences, we utilized VOSviewer 1.6.13 software (Leiden University, Leiden, the Netherlands).

## 3. Results and discussion

### 3.1. Characteristics of the published literature

For studies on RGS16, records found in total from 1995 to 2022, a total of 322, to avoid duplication, we used duplicates removal in CiteSpace,^[14]^ Of the 290 studies, 259 (89.31%) articles; 13 (4.48%) reviews; 33 (11.37%) meeting abstracts and 5 (1.72%) proceedings were included.

**Figure 2.**
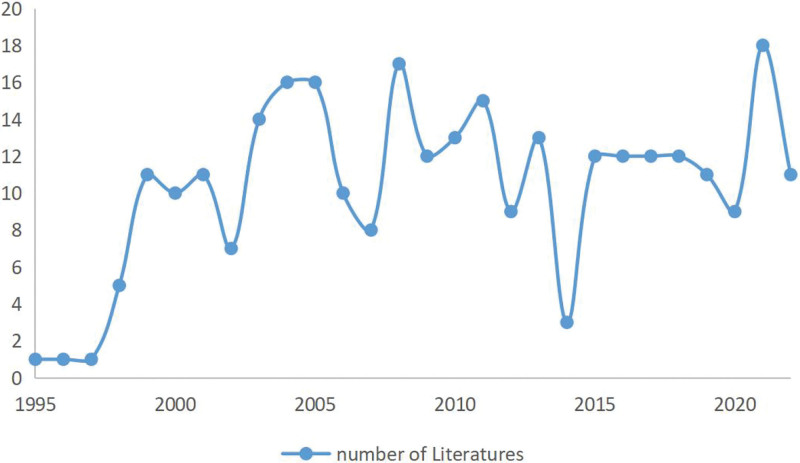
Literature publication statistics for the period 1995–2022.

The year of publication of the 290 articles is shown in Figure 2. 1950 was the first year of the study, and only 1 article entitled “G protein subunit G alpha 16 expressions is restricted to progenitor B cells during human B-cell differentiation.”^[8]^ Then, until 1998, the number of publications remained below 5 per year. From 1999 onward, the number of published articles per year remained above 10 but never exceeded 20. This indicates, from a certain perspective, that the research on RGS16 has not been very hot since its appearance in 1995, but it has been maintained continuously without interruption.

### 3.2. Collaboration between countries/regions

**Figure 3.**
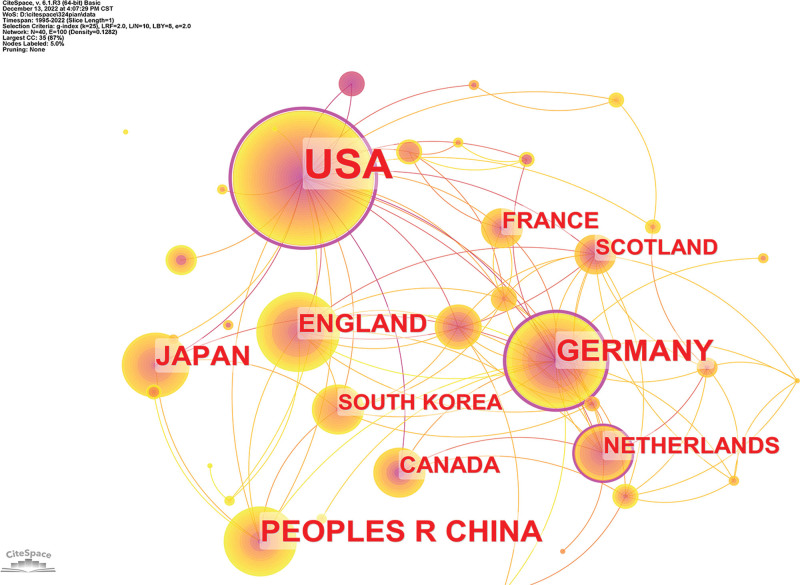
Distribution of cooperation between countries/regions. The nodes represent different countries or regions, and the size of the nodes indicates the volume of posts. And the lines connecting different countries or regions represent collaborations between them, and the thickness of the line represents the number of collaborative postings. The colors of the nodes and lines represent the different years. From 1995 to 2022, the color changes from purple to yellow. Nodes with purple circles denote high mediated centrality (0.1).

Running CiteSpace, we obtained a map with 40 nodes and 100 lines (Fig. 3); this map can help researchers understand if there are people elsewhere doing similar research to themselves and help establish collaborations with them. The RGS16 studies were distributed in 40 countries/regions, and the top 10 countries/regions published 164 papers, accounting for 56.5% of the total (Table 1). The US has the most RGS16 research with a high number of 147 and its centrality of 0.78, indicating that the US is in the leading position in RGS16 research, although China has the second highest number of publications, its Centrality is only 0.09, representing that its research is not in the center. The nearest countries that have done RGS16 research are Saudi Arabia, India, and Georgia, but they have only 4 articles, and the cluster analysis (Supplementary Fig. 1, http://links.lww.com/MD/L398) shows that their research areas are concentrated in RGS protein.

**Table 1:** Characteristics of the 10 cooperating countries/regions.

Rank	Publication	Centrality	Publication Year	Country/Region
1	147	0.78	1996	USA
2	32	0.09	1999	Peoples R China
3	31	0.44	1995	Germany
4	23	0.00	2001	Japan
5	17	0.06	2002	England
6	13	0.10	2001	Netherlands
7	13	0.00	2000	France
8	13	0.01	1998	Canada
9	11	0.01	2006	South Korea
10	11	0.07	2001	Scotland

### 3.3. Cooperation among organizations

**Figure 4.**
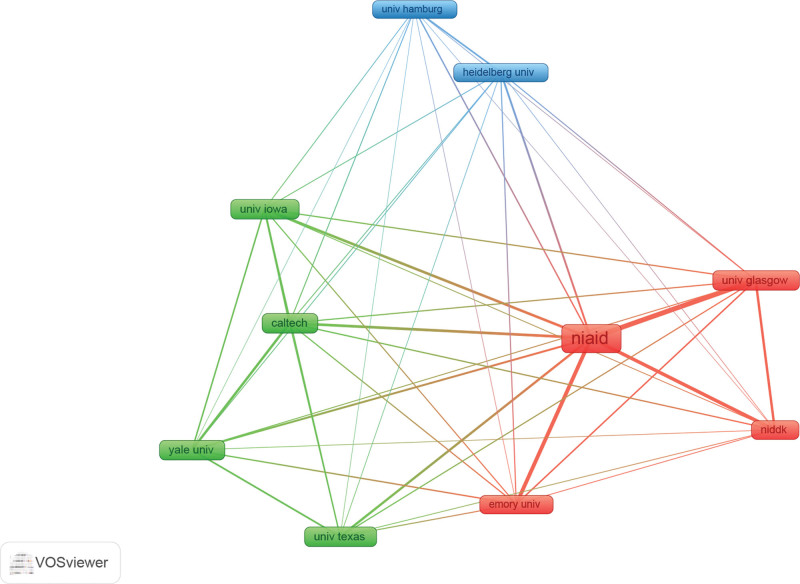
Distribution of collaboration among research institutions.

We used VOS viewers by selecting the Minimum number of documents of an organization = 5. Of the 482 organizations, we get 10 major institutional nodes. The Research Center of Allergy and Infectious Diseases (NIAID), the University of Glasgow, the US Department of Health Center for Kidney Research in the Digestive System of Diabetes (Niddk), the California Institute of Technology, Emory University, Tallinn University of Technology, the University of Hamburg, the University of Texas University of Texas, the University of Heidelberg, and Yale University are a few of these institutions. In Figure 4, we can see more clearly the clustering relationships between the institutions. They are more closely connected. There are 3 colors in the figure, representing the 3 clusters in the institution. Active collaboration usually exists in the same cluster. Connected lines can also exist between different clusters, which means that there are also collaborations between them but with different main research directions from each other.

In the superimposed plot (Supplementary Fig. 2, http://links.lww.com/MD/L399), the size of the text and the thickness of the connecting lines together indicate whether the keyword is dominant in the study data sample. The more yellow-leaning text box color represents the more recent studies closer to 2022. The distance between keywords represents a stronger collaborative relationship with other institutions, and conversely, a weaker co-occurrence relationship. Emory University color is closest to yellow, representing that Emory University is the most recent of all institutions with the most recent results in the RGS16 study.

In the CiteSpace analysis, the top 10 institutions in terms of volume and centrality are shown in Table 2. NIAID has the highest volume of articles (18), but its centrality is only 0.09, which is not enough to be considered a major research team among all institutions. University of California, San Diego, on the other hand, has far fewer publications than NIAID, but it has a leading position than NIAID.

**Table 2:** Volume and centrality of articles issued by the top 10 institutions.

Rank	Publication	Centrality	Publication Year	Organization
1	18	0.09	1999	NIAID
2	8	0.05	1999	CALTECH
3	8	0.00	1998	University of Iowa
4	7	0.01	2001	University of Glasgow
5	6	0.03	2003	Heidelberg University
6	6	0.00	2000	Emory University
7	5	0.00	1999	Yale University
8	5	0.10	2000	University of California San Diego
9	5	0.00	1999	Univeristy of Texas
10	5	0.03	2001	Harvard University

**Figure 5.**
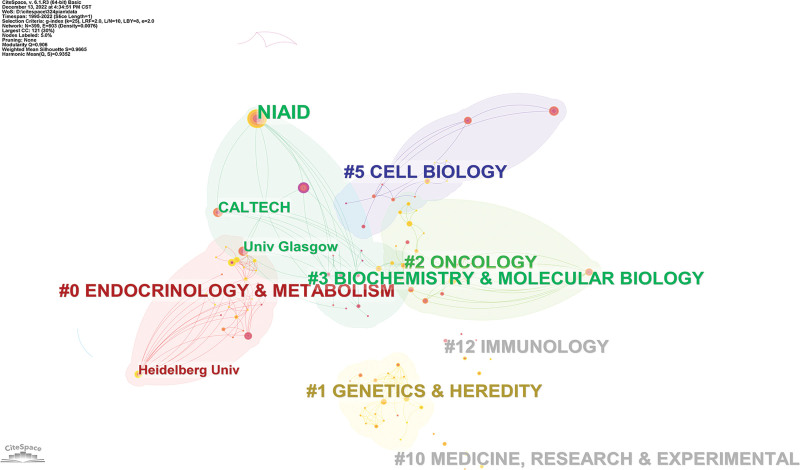
Institutional clustering analysis.

In the timeline analysis, we found a total of 7 clusters (Fig. 5, Supplementary Fig. 3, http://links.lww.com/MD/L402). We looked for the 10 most researched institutions one by one. We found that Heidelberg University is mainly in the field of Endocrinology and metabolism; Harvard University is in oncology; NIAID, Caltech, University of Glasgow, University of California, San Diego is in biochemistry and molecular biology biochemistry and molecular biology; Yale University in addition, there are 2 other institutions not found in the above 7 clusters, including Emory University, University of Iowa, which are considered to be removed in some screening conditions.

Heidelberg University has been extensively studied on the expression of RGS16 protein in cardiomyocytes. The 2 most frequently cited articles analyzed the effects of endothelin-1 (ET-1), sphingo 16-phosphate (S1P), and IL-1β on the expression of RGS16 protein through mouse and cell experiments.^[15,16]^ Harvard University is more focused on genome research.^[17]^ Emory University and University of California, San Diego, study membrane channels and receptors.^[18]^ CALTECH studies the molecular structure of RGS16.^[19]^ NIAID,^[20]^ research interests focus on biochemistry, immunology, cardio-cardiovascular system, and cell biology, more specifically, focus on RGS16 signal transduction mode, and platelet regulation. University of Glasgow, in close collaboration with NIAID, suggested that palmitoylation may affect the activity and function of RGS16 protein.

### 3.4. Keyword co-occurrence

We conducted a co-occurrence analysis of keywords, and since some non-RGS16-related studies may be included when including data from the literature, we adjusted the selection criteria, after top *N* = 100; we could find that RGS16 involved 115 keywords with a total of 491 keywords connected. It has a harmonic mean (*Q, S*) of 0.9882, weighted modularity *Q* of 0.9808 and mean silhouette *S* of 0.9956. This represents a considerable plausibility and credibility value for our analysis. Then, the 20 keywords with the highest repeatability in each year were screened (Table 3). It is also the frequency of occurrence of all keywords >10. We found that the keywords centrality of expression, regulator, and activation are >0.3, which represents that they have higher centrality compared with other keywords and can be better called the keyword co-occurrence network from 1995 to 2022 is presented in Supplementary Figure 4, http://links.lww.com/MD/L404. In this diagram, each node stands for a term, and the more noticeable the yellow, the more innovative the research is. Among the top 20 keywords, expression, regulator, and gene expression are relatively new, but we still cannot visualize the research timeline of each keyword and whether it is still a research hotspot, for which we need to perform cluster analysis and burst analysis to obtain which can be used as a research hotspot at present.

**Table 3:** Keywords with frequencies >10 in keyword co-occurrence.

Rank	Count	Centrality	Year	Keywords	Clustering
1	64	0.32	2001	expression	#4
2	45	0.21	1998	GTPase-activating protein	#0
3	41	0.34	1999	Regulator	#2
4	36	0.22	2005	Gene expression	#0
5	31	0.16	1998	RGS protein	#0
6	26	0.16	1999	Gene	#2
7	26	0.08	1999	Heterotrimeric g-protein	#1
8	26	0.08	1998	Alpha subunit	#1
9	25	0.12	2000	Receptor	#0
10	21	0.30	2000	Activation	#3
11	20	0.18	2001	g-Protein	#5
12	20	0.10	1998	Family	#0
13	15	0.05	2000	Domain	#1
14	13	0.01	1999	Gaip	#1
15	12	0.05	2002	Messenger rna	#2
16	12	0.10	1998	Adenylyl cyclase	#2
17	12	0.01	2005	Protein	#0
18	11	0.01	2004	Identification	#0
19	11	0.01	1999	Phospholipase c	#1
20	10	0.02	1999	Inhibition	#2

We clustered by discipline and obtained 6 clusters after analysis (Supplementary Fig. 5, http://links.lww.com/MD/L407), each representing a subject category, and we obtained the subject categories clusters for the top 20 keywords. #0 CELL BIOLOGY; #1 BOCHEMISTRY & MOLECULAR BIOLOGY; #2 PSYCHIATRY; #3 ENDOCRINOLOGY & METABOLIS; #4 BIOTECHNOLOGY & APPLIED MICROBIOLOGY; #5 PERIPHERAL VASCULAR DISEASE. only #4 BIOTECHNOLOGY & APPLIED MICROBIOLOGY is still under investigation, with preexisting studies centered on expression, mice lacking,^[21–23]^ in vitro,^[24,25]^ mid-term around pathogenesis,^[26,27]^ cancer,^[28,29]^ Currently around inflammation,^[28,30]^ injury,^[31]^ antibody,^[32,33]^ release,^[34,35]^ protein signaling 16,^[33,36]^ the latest is migration.^[37,38]^ The most recently researched topic in the most researched discipline is corpus luteum. while clustering by keyword, 6 clustering areas were found (Supplementary Fig. 6, http://links.lww.com/MD/L411), only #4 pig is still under continued research, which contains an expression, differentiation,^[39,40]^ RGS4, mice lacking,^[21]^ cancer, cell, NF-κB, release, antibody, inflammation, injure, migration, and other keywords.

In the analysis of burst intensity when clustering by discipline category (Supplementary Fig. 7, http://links.lww.com/MD/L413), adjusting burstness *y* value = 0.5 and view = 30, we found that expression (6.39) and gene expression (6.85) was still in the burst state. Although #0 is not in the continuation state in the time graph, its core keyword gene expression (6.85) is still in the outbreak state.

Therefore, we believe that #4 BIOTECHNOLOGY & APPLIED MICROBIOLOGY is still a popular area for research, and expression and gene expression are still hot spots for research.

We used VOS viewers to conduct co-occurrence and density analysis of keywords to determine which keywords have received the most recent research (Supplementary Fig. 8, http://links.lww.com/MD/L414, and Supplementary Fig. 9, http://links.lww.com/MD/L418), where the node size represents the frequency of co-occurrence, the linking line shows that the research between keywords has significance, the thickness of the connecting line represents the size of importance, the same color represents that it is in the same field of research, and the yellow area indicates that the study time is getting closer to the current. We can find protein signaling 16, genetics, stem cells, migration, cancer, stress, mete-analysis, mice, etc are perhaps the current emerging research areas.

### 3.5. Analysis of citing journals

**Figure 6.**
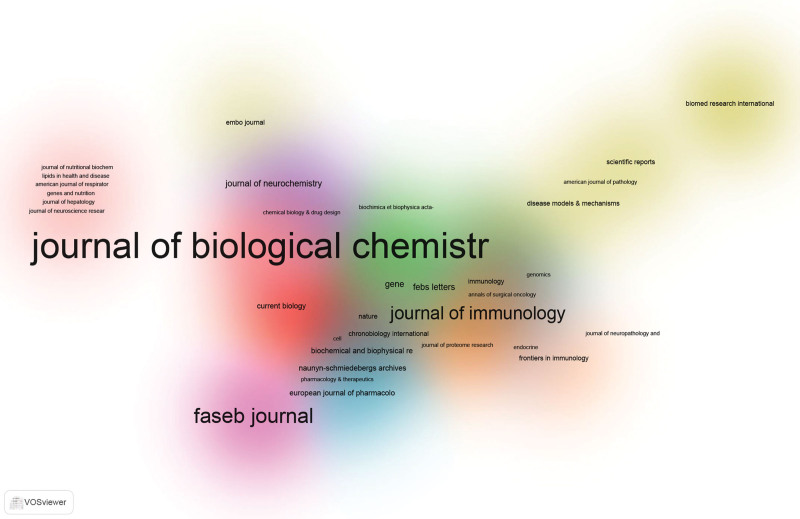
Density graph of co-cited journals. The color represents the references of the 290 articles we selected, in which the fields of study are the same, and the text size represents the frequency of citations; the higher the frequency, the larger the font.

**Figure 7.**
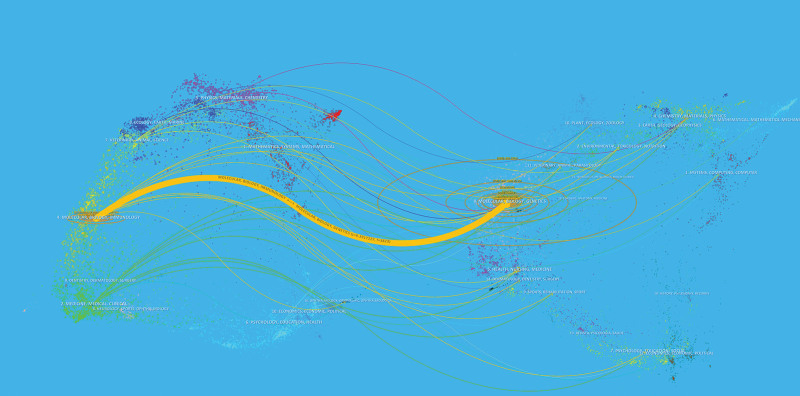
Dual map overlay of co-cited journals.

**Figure 8.**
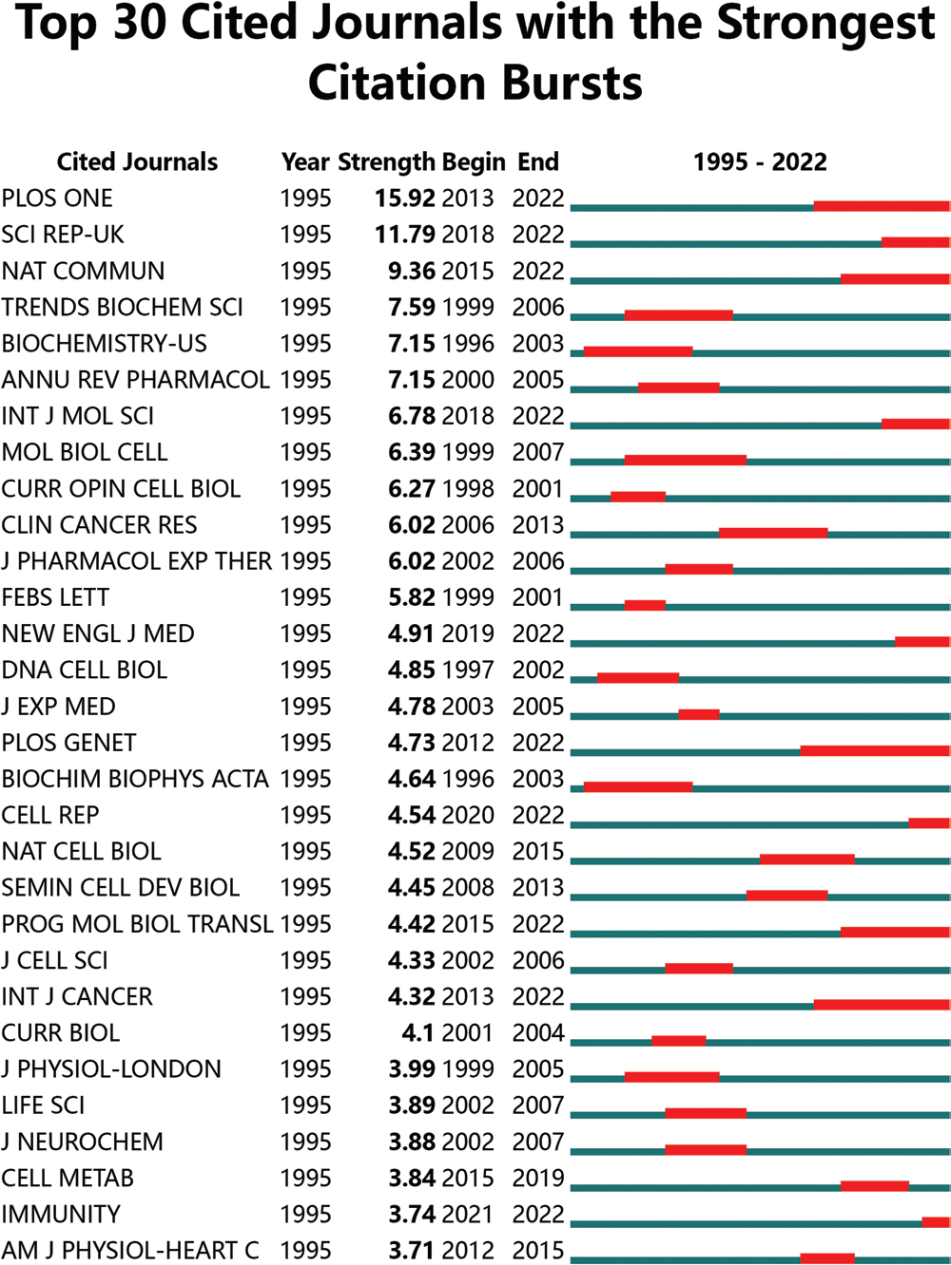
Co-citation journal outbreak values.

“Core journals” are very often top journals with a high reference frequency. Using VOS viewer software, we found that 179 academic journals published articles on RGS16 studies. About 8 journals published more than 5 studies, with 93 publications (Table 4), accounting for 32.06% of all publications. The journal of biological chemistry had the most papers (*n* = 31), followed by the journal of immunology (*n* = 16) and the FASEB journal (*n* = 13). A total of 1840 sources were cited and the top 8 most cited journals with > 180 citations are listed in Table 4. Their density plots are presented in Figure 6, which shows the relatively concentrated distribution of citations. As a result, the Journal of Biological Chemistry (IF = 5.485) is the “core journal” in the subject of RGS16. The subject allocation of academic publications is represented by a double map of journals (Fig. 7). The left side shows cited journals, the right side shows citation relationships, and the colored paths show citation links. Showing only one main reference path from Molecular, Biology, Immunology to Molecular, Biology, and Genetics. In the Burst analysis (Fig. 8), we found that the journals that are still showing an outbreak include Plos One, Sci Rep-UK, Nat Commun, Int J Mol Sci, New Engl J Med, Plos Genet, Cell Rep, Prog Mol Biol Transl, Int J Cancer, and Immunity. This means that these 11 journals have been highly cited in recent years and are important journals in the field of RGS16 research in recent years.

**Table 4:** 10 highly cited journals IF issued in 2021.

Rank	Journal	Count	JCR (2021)	IF (2021)	Citing journals	Number of citations	JCR (2021)	IF (2021)
1	J. Biol. Chem	31	Q2	5.485	J. Biol. Chem	1609	Q2	5.485
2	FASEB J	16	Q2	5.834	Proc. Natl. Acad. Sci	759	Q1	12.779
3	J. Immunol	13	Q2	5.43	nature	523	Q1	69.504
4	plos one	9	Q2	3.752	cell	372	Q1	66.85
5	Mol. Pharmacol	7	Q2	4.058	J. Immunol	316	Q2	5.43
6	Proc. Natl. Acad. Sci	7	Q1	12.779	science	302	Q1	63.832
7	Cell. Signal	5	Q2	4.85	J. Neurosci	185	Q1	6.709
8	Nat. Commun	5	Q1	17.694	Mol. Cell. Biochem	184	Q3	3.842

### 3.6. Literature co-citation analysis

Of the 14,560 cited references, we used the CiteSpace default settings and 810 were considered as co-cited. Table 5 selects the top 10 co-cited references that were cited at least 22 times. The most often referenced reference is an article titled “Structure of RGS4 bound to AlF4--activated G (i alpha1): stabilization of the transition state for GTP hydrolysis,”^[41]^ published in Cell by John J. G. Tesmer et al in 1997, with a relatively high centrality [=0.16]. Another article with high centrality is “GTPase-activating proteins for heterotrimeric G proteins: regulators of G protein signaling (RGS) and RGS-Like Proteins,”^[3]^ with a centrality of 0.21. This means that they are perhaps the core literature in the field of RGS16 research. We also screened 10 high and high school cardinalities out of 810 co-citations, which are presented in Table 6. Despite their low citation frequency, they all hold a relatively important place in the field of RGS16 research.

**Table 5:** Collection of top 10 co-cited highly cited literature.

Rank	Citation count	Centrality	Publication year	Title
1	30	0.16	1997	Structure of RGS4 bound to AlF4 2-activated Gia1: stabilization of the transition state for GTP hydrolysis
2	29	0.21	2000	GTPase-activating proteins FOR heterotrimeric G-proteins: regulators of G-protein signaling [RGS] and RGS-like proteins
3	25	0.05	1996	GAIP and RGS4 Are GTPase-activating proteins for the Gi subfamily of G-Protein a subunits
4	25	0.02	1996	RGS family members: GTPase-activating proteins for heterotrimeric G-protein α-subunits
5	24	0.25	1996	EGL-10 Regulates G-protein signaling in the C. elegans nervous system and shares a conserved domain with many mammalian proteins
6	23	0.09	2002	Cellular regulation of RGS proteins: modulators and integrators of G-protein signaling
7	23	0.06	1997	Characterization of a novel mammalian RGS protein that binds to Ga proteins and inhibits pheromone signaling in yeast
8	22	0.02	1997	RGS Proteins and Signaling by Heterotrimeric G Proteins
9	22	0.00	1996	RGS-r, a retinal-specific RGS protein, binds an intermediate conformation of transducin and enhances recycling
10	21	0.08	1996	Inhibition of G-protein-mediated MAP kinase activation by a new mammalian gene family

**Table 6:** Co-referenced high-centricity set of literature.

Rank	Citation count	Centrality	Publication year	Title
1	24	0.25	1996	EGL-10 regulates G-protein signaling in the C. elegans nervous system and shares a conserved domain with many mammalian proteins
2	6	0.25	2008	Rgs5 targeting leads to chronic low blood pressure and a lean body habitus
3	5	0.23	1999	Antagonism between G[o]alpha and G[q]alpha in Caenorhabditis elegans: the RGS protein EAT-16 is necessary for G[o]alpha signaling and regulates G[q]alpha activity
4	29	0.21	2000	GTPase-activating proteins for heterotrimeric G-proteins: regulators of G protein signaling (RGS) and RGS-like proteins
5	6	0.19	2000	RGS4 binds to membranes through an amphipathic a-helix
6	8	0.17	2003	Regulators of G-protein signaling: multifunctional proteins with impact on signaling in the cardiovascular system
7	4	0.17	1996	G Proteins are required for spatial orientation of early cell cleavages in C. elegans embryos
8	30	0.16	1997	Structure of RGS4 Bound to AlF4 2-activated gia1: stabilization of the transition state for GTP hydrolysis
9	20	0.16	2000	GTPase-Activating Proteins for Heterotrimeric G Proteins: Regulators of G Protein Signaling (RGS) and RGS-Like Proteins
10	6	0.15	2006	RGS proteins have a signaling complex: interactions between RGS proteins and GPCRs, effectors, and auxiliary proteins

Based on the timeline analysis (Supplementary Fig. 10, http://links.lww.com/MD/L419, and Supplementary Fig. 11, http://links.lww.com/MD/L420), we can see that all the reference themes are mainly contained in 14 clusters. Among them, the disciplinary perspective #1, PHARMACOLOGY & PHARMACY, and the keyword perspective #1 inflammation are still under continued research, which represents that it may be an emerging field.

A burst reference is cited relatively frequently over some time. By adjusting burstness ɣ = 0.5 and minimum duration = 1, we discovered 129 references that were determined to be citing bursts, and we provided the top 20 (Supplementary Fig. 12, http://links.lww.com/MD/L421). The strongest burst (intensity = 7.73) occurred in a paper entitled “GTPase-activating proteins for heterotrimeric G proteins: Regulators of G protein signaling (RGS) and RGS-like proteins,”^[3]^ published by Ross et al in Annu Rev Biochem in 2000, with a citation burst from 2001 to 2008. In addition, there is another reference still in outbreak status. It is a review by Suurväli et al published in Scand J Immunol in 2015, entitled “RGS16 restricts the pro-inflammatory response of monocytes.”^[42]^ In addition to the 20 papers we screened, there is another paper entitled “The regulator of G-protein signaling 18 regulates platelet aggregation, hemostasis, and thrombosis”^[43]^ that is also in outbreak status, although its outbreak value is relatively small (outbreak value = 2.85) and the outbreak years are 2016–2022.

### 3.7. Author analysis

The idea of author co-citation, which has been widely utilized to judge scientific competency and relevance, demonstrates the proximity and importance of the author’s specific research.

RGS16 research included 762 authors in totality 65 of them published at least 2 publications (Table 7, Supplementary Fig. 13, http://links.lww.com/MD/L423). Drury KM published the most RGS16-related articles (*n* = 15), followed by Wieland. In the attached and Supplementary Figure 14, http://links.lww.com/MD/L425, we can see that there is no direct linkage between Druey and Wieland. This means that they do not have a collaborative relationship, but each leads a different team to conduct their research. To understand the collaboration between them and their respective research areas, a cluster analysis is necessary (Supplementary Fig. 17, http://links.lww.com/MD/L430, and Supplementary Fig. 18, http://links.lww.com/MD/L433). In the timeline study (Supplementary Fig. 15, http://links.lww.com/MD/L426, and Supplementary Fig. 16, http://links.lww.com/MD/L427), we can also see that both of them have been researching for basically the same amount of time, but Druey mainly conducts RGS proteins research, while Wieland mainly researches in the liver field.

**Table 7:** Author and co-cited author publications.

Rank	Author	Number of articles	H-index	Author cited	Number of citations	H-index
1	Druey KM	15	35	Berman DM	68	47
2	Wieland T	7	44	Druey KM	63	35
3	Artemyev NO	5	33	Ross EM	55	53
4	Chen CK	5	17	Dohlman HG	46	48
5	Lin SC	5	43	Heximer SP	43	31
6	JONES TLZ	4	29	KOELLE MR	42	24
7	Chen CH	4	5	Chen CH	40	5
8	Milligan G	4	83	Hepler JR	38	44
9	Hiol A	3	14	Tesmer JJG	36	17
10	Alewijnse AE	3	26	Chen CK	36	17

Out of 8766 co-cited authors, only 49 authors had more than 10 co-cited articles, with Berman, having the highest number of articles (*n* = 106; Table 7). Their density maps are Supplementary Figure 19, http://links.lww.com/MD/L434, and Supplementary Figure 20, http://links.lww.com/MD/L436, highlighting the writers with the highest frequency of citations. The hotter the color, the more citations there are. Berman has the most co-citations. One of his articles is a high-frequency co-citation and is entitled “GAIP and RGS4 are GTPase-activating proteins for the Gi subfamily of G protein alpha subunits.”^[44]^

As previously stated, the RGS16 work was greatly influenced by the team of Berman, and other writers from the Department of Pharmacology and the University of Texas Southwestern Medical Center.

## 4. Discussion

### 4.1. General information

According to the WoSCC database from 1995 to 2022, 618 authors from 399 institutions in 40 countries/regions have published 290 RGS16 articles in 179 academic journals, with a cited literature volume of 14,560.

The first RGS16-related literature^[8]^ was published in April 1995, and since then, the research on RGS16 has been gradually generated. Since 1999, most of the RGS16-related articles published each year have been maintained at more than 10, but never more than 20. We believe that RGS16 research is not very hot, but researchers have maintained an interest in it.

In the analysis of countries/regions and institutions, we mainly judge whether they are in the leading position by the number of publications and centrality, and we generally believe that the countries/regions with the most publications are probably the core research countries/regions, and the nodes with high centrality [≥0.10] represent the countries/regions with “bridge” effect.^[33,34,45,46]^ According to the results, the 10 countries with the most published RGS16-related literature include the USA, P.R. China, Germany, Japan, England, the Netherlands, France, Canada, South Korea, and Scotland. Among 10 institutions with the most published research papers, 3 are in USA. However, except for the USA (Centrality), 2 are in Germany, and 1 each in the UK and Estonia. However, the centrality of all 7 countries/regions except the USA (Centrality = 0.78), Germany (Centrality = 0.44), and the Netherlands (Centrality = 0.10) is <0.1, indicating that USA and Germany, the Netherlands may be dominance in RGS16 research. In addition, we can see based on VOS viewers that there is relatively close cooperation among various countries/regions and institutions in terms of research density. The density graph is in the supplementary.

Among the top ten writers and co-cited writers, Druey not only produced the most RGS16-related publications, but he was also 1 of the top 2 co-cited authors, demonstrating his excellent contribution to RGS16 research. One of his articles is also in the top ten most quoted. The RGS family members also significantly decrease MAP kinase function by mammalian G-protein-linked receptors, confirming the existence and relevance of an SST2-like desensitization pathway in mammalian cells, according to the report.^[47]^ Since its publication in 1996, it has been co-cited as many as 21 times and is in the top 10 citations in this study. From 1997 to 2002, there was a strong explosion of this reference. Notably, the most cited author, Berman^[44]^ published a top 10 co-cited article. The genetic evidence presented implies that RGS proteins block G-protein-mediated activation at the receptor-G-protein interaction or at the level of the G-proteins alpha subunit itself.

Among the journals and co-cited literature among the 290 publications we included, 8 journals published more than 5 studies, with a total of 93 publications, accounting for 32.06% of all publications. Among them, the Journal of Biological Chemistry published 31 RGS16-related studies, which is the most published among the 8 journals and the most cited among the cited journals. This indicates that it plays a key role in publishing RGS16 studies. Two of the critically important papers were among the top 10 highly cited papers in terms of co-citations, both with more than 20 co-citations, under the names “Characterization of a Novel Mammalian RGS Protein That Binds to Ga Proteins and Inhibits Pheromone Signaling in Yeast,”^[48]^ and “RGS Proteins and Signaling by Heterotrimeric G Proteins.”^[45]^ In the journal bitmap analysis, the main citation paths indicating RGS16 research were from Molecular, Biology, Immunology to Molecular, Biology, Genetics.

### 4.2. Current situation and hotspots

The visualization of literature data allows us to keep abreast of the progress in the research field. Keyword co-occurrence is used to depict hotspots in the area of study, clustering, and timeline views are used to show the evolution of new hotspots, and references clustering and citation burst are used to illustrate emergent issues in the discipline.^[46]^ This study attempts to use keyword co-occurrence, keywords in terms of domain type and keywords, clustering and timeline analysis; keyword burst, co-citation co-occurrence, clustering, and reference burst to analyze which are the hot and emerging studies in RGS16 research.

The RGS16 protein structure was largely the subject of research in the important literature we gleaned from the screening, which has already been more thoroughly covered in 1 body of literature.^[5]^ This does not imply that the work we have done has been merely repeated, and the primary focus of our research is on more recent findings, specifically the potential disease applications of RGS16. Future RGS16 researchers will be presented with the current state of the field and its hotspots, and we anticipate that some of the issues raised will merit further investigation. We have included the prospective illness investigations below.

#### 4.2..1. RGS16 protein structure.

Based on the 20 high-frequency citations obtained from the reference co-occurrence analysis as well as the high-centricity literature, the themes revolve around RGS protein structure, which is often cited in subsequent articles as the beginning of RGS protein and RGS16 research, and are the focus and key of RGS16 research, but are not the current hot spots that our study focuses on. In the year 2000, in a review, Ross^[3]^ was written. The RGS proteins are linked by a 130 amino acid residue long conserved RGS domain. Other domains may help with affinity and/or choice for G-protein targets in addition to the RGS domain, which is able to bind G subunits and speeding GTP hydrolysis. The brain expresses almost all RGS genes. Nonneural tissues and cells also regulate many RGS mRNAs. RGS16 is expressed in colon cancer cells, RGS2 is expressed in TC3 insulinoma cells, as well as RGS16 is expressed in T-lymphocytes, ventricular cardiomyocytes, and the liver.^[1]^ Watson^[49]^ considers RGS proteins are likely to have a variety of physiologic functions in mammalian cells. Because they may provide GAP activity, controlling the expression of RGS family members may therefore provide a versatile means of regulating G-protein signaling pathways for a variety of physiological imposes. Combinatorial, spatial, temporal, and developmental modulation of RGS function definitely could influence the strength, duration, location, and cell-type specificity of G-protein signaling, according to Dohlman results.^[45]^

#### 4.2..2. Potential disease research of RGS16.

According to our results, biological applications of RGS16 are currently a hot area of RGS16 research, including inflammation,^[30]^ cancer,^[28]^ ulcerative colitis,^[38]^ metabolic acidosis,^[34]^ platelet activation, and thrombosis.^[50,51]^ We discuss allergic and irritant contact dermatitis and schizophrenia below.

The role of RGS16 in allergic and irritant contact dermatitis is currently only used as a marker to distinguish between the 2 diseases. According to Fortino^[30]^, it is considered that Random Forest Classification identified RGS16 and 21 different proteins as potential biomarkers to distinguish allergic and irritant contact dermatitis in human skin. Considering, on the other hand, RGS16 has great potential in inflammation therapy. In particular, the therapeutic potential through monocytes, mast cells, and allergens can interact with and activate mast cells, T-lymphocytes, and mononuclear phagocytes, leading to the secretion of cytokines and other inflammatory substances. The spectrum of known mediators released after allergen exposure has been greatly expanded. These mediators recruit and activate neutrophils, monocytes, basophils, and eosinophils in a vicious cycle that leads to further inflammation and tissue destruction.^[52]^ The regulator of G-protein signaling 16 (RGS16) has been identified as a key factor of G-protein-mediated activation in lymphocytes, modulating inflammatory and survival responses of various cell types. Overall, RGS16 attenuates the inflammatory response of monocytes and is likely involved in complex regulatory loops.^[42]^ Although the structure of RGS16 and the signal transduction mechanism have been well-studied.^[45,53]^ However, its research and application in inflammation are just beginning to emerge, and further research is needed on whether it can serve as a target for allergic and irritant contact dermatitis treatment and whether it has greater potential for validation.

Another noteworthy point is that, based on gene expression meta-analysis,^[54]^ Schizophrenia patients RGS16 reveal up-regulation of RGS16 in Brodmann Area 10. Through VOS viewers, we can also see that meta-analysis is 1 of the more novel research areas in RGS16 research. On one hand, almost all RGS genes are expressed in the brain. Many RGS mRNAs are also regulated in nonneural cells and tissues.^[3]^ On the other hand, Campbell^[55]^ organized the clinical antipsychotic trials of intervention effectiveness. Although these analyses are exploratory and replication is required, these data suggest a possible role for multiple RGS proteins in schizophrenia. Almost all RGS genes are expressed in the brain. Many RGS mRNAs are also regulated in nonneural cells and tissues.^[49,56]^ Perhaps RGS16 will have something to do with schizophrenia.

RGS16 protein is expressed in a variety of tissues and is associated with a variety of cancers, but the application of RGS16 in the field of cancer is not perfect, and most of the current studies believe that RGS16 can be used as a biomarker for the diagnosis and prognosis of a variety of cancers. In particular, RGS16 has been suggested as a new therapeutic target for breast cancer.^[5]^ Recent studies mainly focus on chronic metabolic acidosis (MET),^[57]^ ovarian cancer,^[58]^ promotes antitumor CD8 T cell exhaustion,^[59]^ bioactive lipid accumulation, and hepatic inflammation.^[60]^ The primary bone cell and primary hepatocyte were mainly used. Although the structure and signal transduction mechanism of RGS16 has been relatively clear, whether it has greater potential needs further study. Based on the study of RGS16 in oncology, we found that there are still new research advances in PATHOLOGY and SURGERY, mainly including signature, PD 1,^[59]^ bone calcium, histone deacetylase inhibitor, gene signature, gemcitabine,^[59]^ hepatocellular carcinoma,^[61]^ and pancreatic ductal adenocarcinoma.

### 4.3. Strengths and limitations

Overall, this is the first data visualization project to carefully examine previous RGS16-related papers.

In comparison to traditional evaluations, data visualization analysis offers fresh and impartial insights into changing research goals and trends.^[62]^ Overall, this is the initial data visualization project to carefully examine previous RGS16-related papers. In comparison to traditional evaluations, data visualization analysis offers fresh and impartial insights into changing research goals and trends.^[63]^ This study will educate the public about the significance of RGS16, give scholars a comprehensive image of RGS16 research, and provide thorough and objective guidance for the future growth of this research field.

Despite the fact that English papers in WoSCC are the most often used source of data in visual analytics and can represent the majority of the information to a certain extent.^[64]^ There will always be flaws in our research. First, screening is undertaken throughout the data recovery process, which inevitably misses RGS16-related material as well as those with minimal significance. Nonetheless, we had to seek to exclude the majority of the irrelevant material. Second, as other research has shown, the data presentation and analysis method is reliant on natural-language processing, which could be biased.^[65]^ Our findings, however, are similar to the most current mainstream review.^[5]^ It also offers more thorough and objective data.

## 5. Conclusion

In summary, RGS16 research results have been steadily increasing in the last decade under worldwide research. The United States may continue to hold a strong position in RGS16 research. Journal of Biological Chemistry has published the most relevant publications, and Druey is the most crucial author in the field of RGS16. The structure of RGS16 has been relatively well studied, and current research hotspots are focused on potential applications in diseases. In particular, inflammation, cancer, ulcerative colitis, metabolic acidosis, platelet activation and thrombosis, allergic and irritant contact dermatitis, and schizophrenia. RGS16 plays an important role in tumor development and is expected to be a potential therapeutic target for regulating tumor process. We hope that our research can provide direction and new insights for the future research of RGS16.

Supplementary Materials: The list of publications used for data visualization studies (subject search results in the SCI-E database) includes the article title, journal name, author, year of publication, volume, and page range. The following is available in the link below.

https://www.webofscience.com/wos/woscc/summary/d300812f-9f49-47c6-97d9-18cb5f9d9d1b-616a9fe9/relevance/1

For other supplementary materials, please refer to the uploaded supplementary documents.

## Author contributions

Data curation: Liu Wenbo.

Writing—original draft: Liu Wenbo.

Methodology: Xie Liangyu.

Software: Lu Zhiyong.

Writing—review & editing: Yu Gongchang, Chen Yuanzhen, Shi Bin.

## Supplementary Material








































