# Supplementary figures and images for: Status and trends of RGS16 based on data visualization analysis: A review
Source: Medicine (Baltimore). 2024 Feb 16;103(7):e36981. doi: 10.1097/MD.0000000000036981 (PMC10869050; doi:10.1097/MD.0000000000036981)

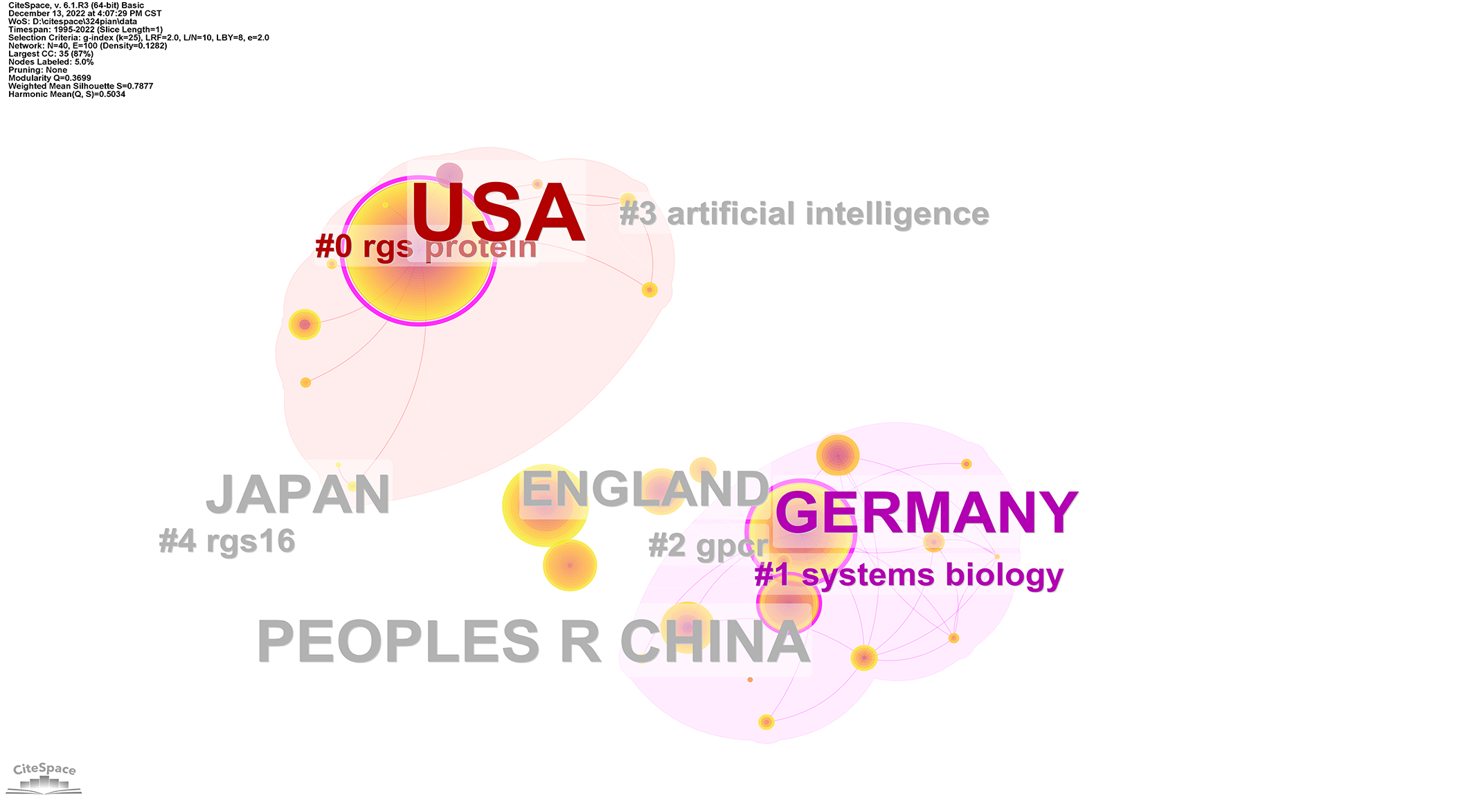

Supplement: Supplementary file 1 [file medi-103-e36981-s001.tif]

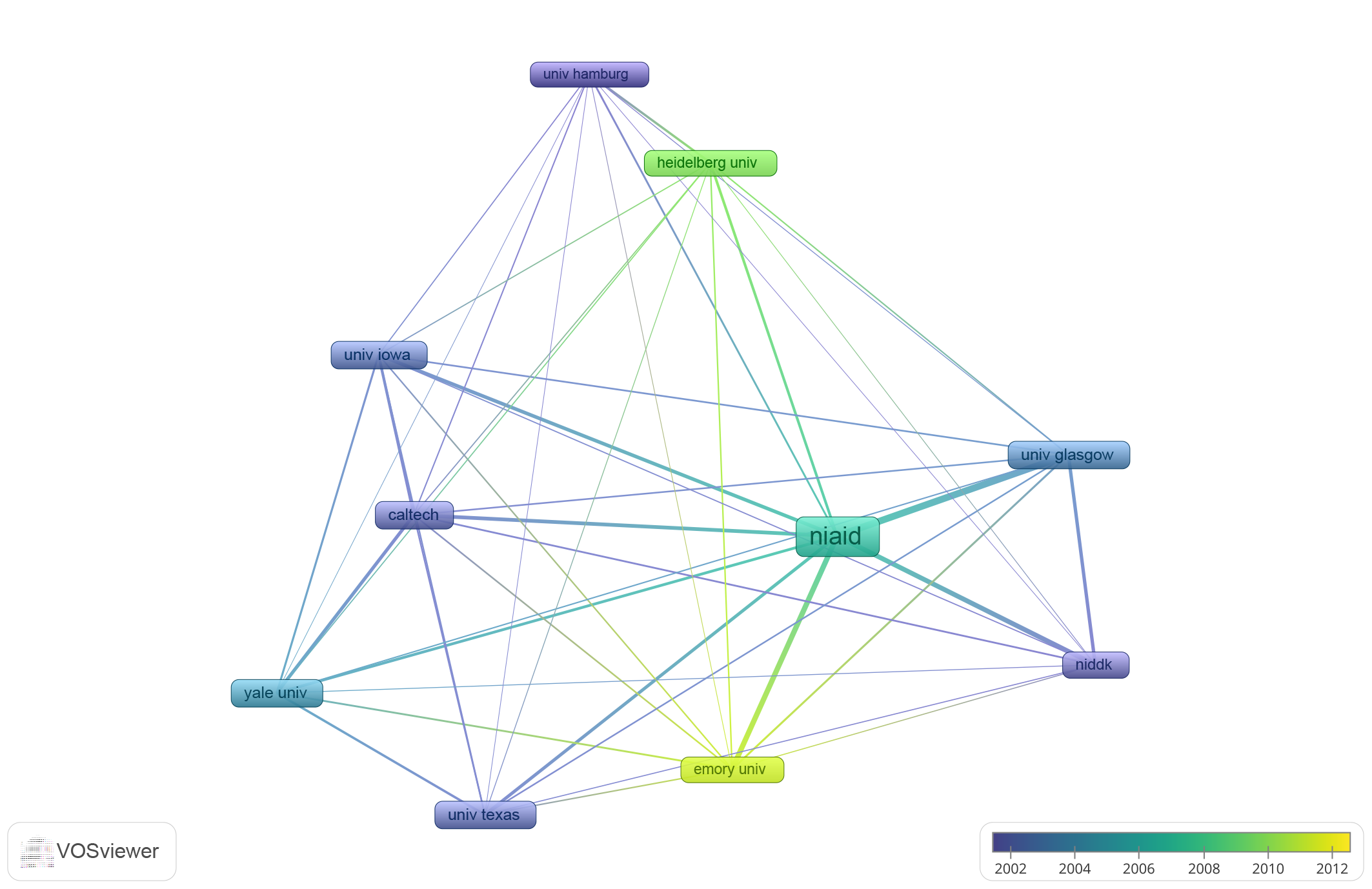

Supplement: Supplementary file 2 [file medi-103-e36981-s002.tif]

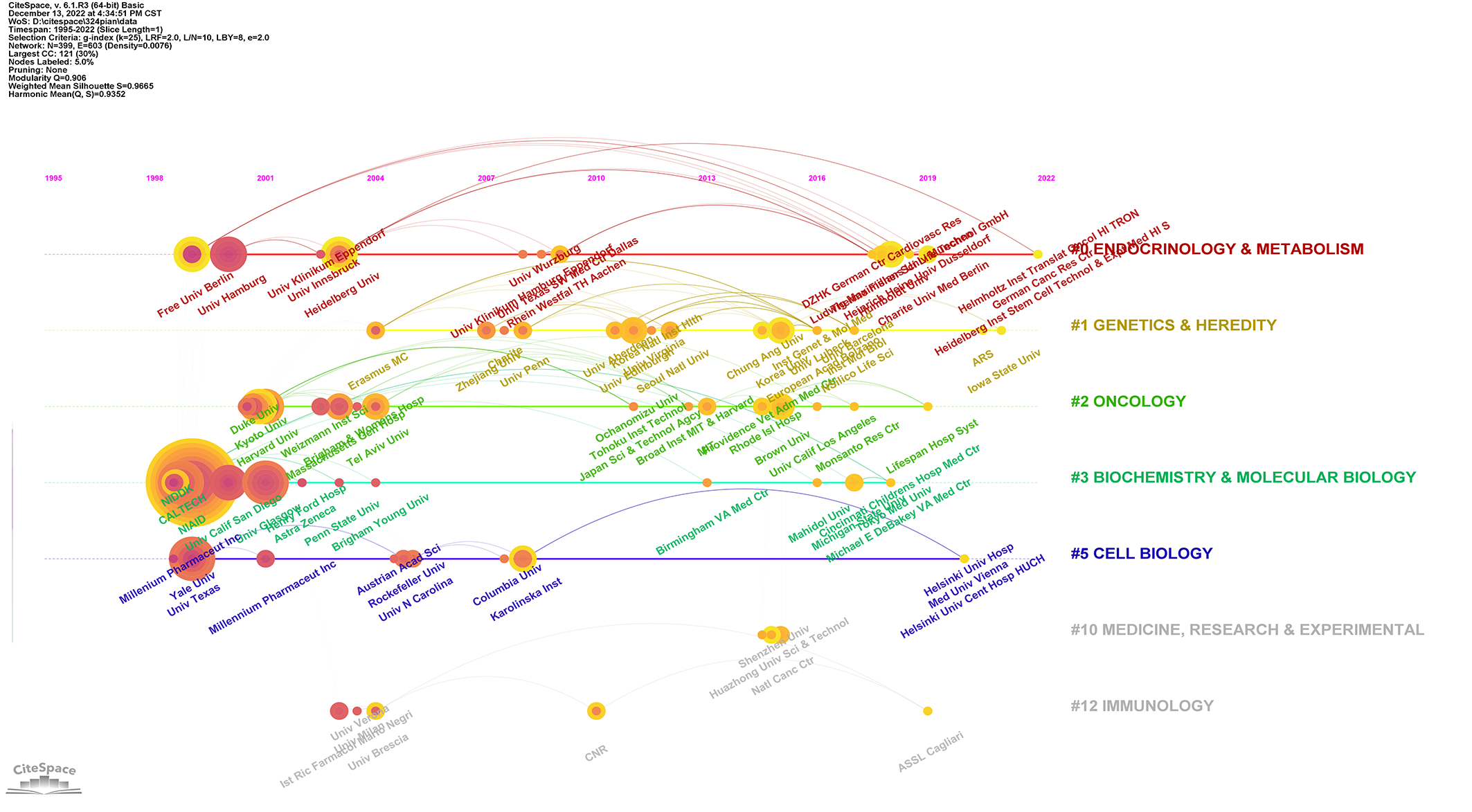

Supplement: Supplementary file 3 [file medi-103-e36981-s003.tif]

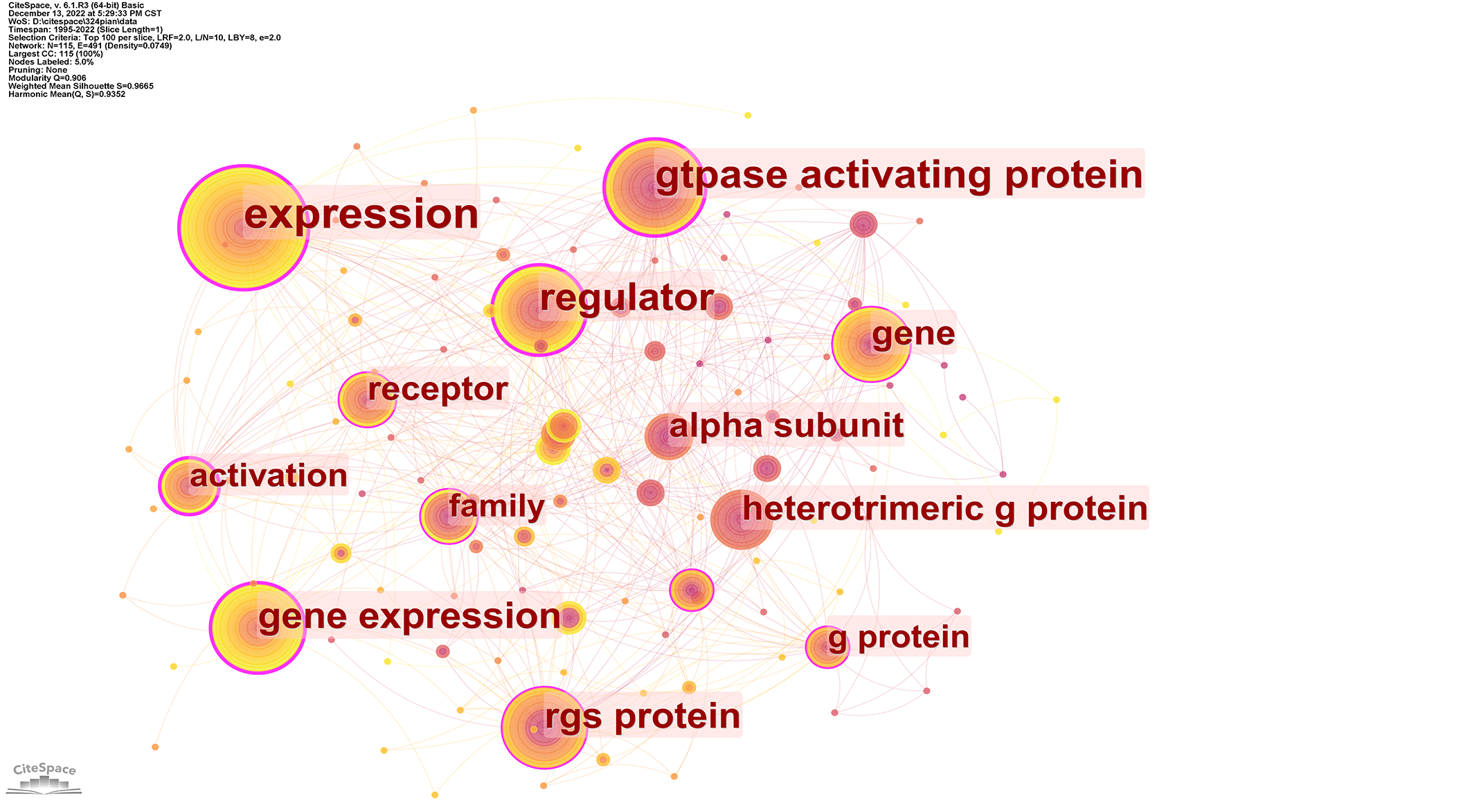

Supplement: Supplementary file 4 [file medi-103-e36981-s004.tif]

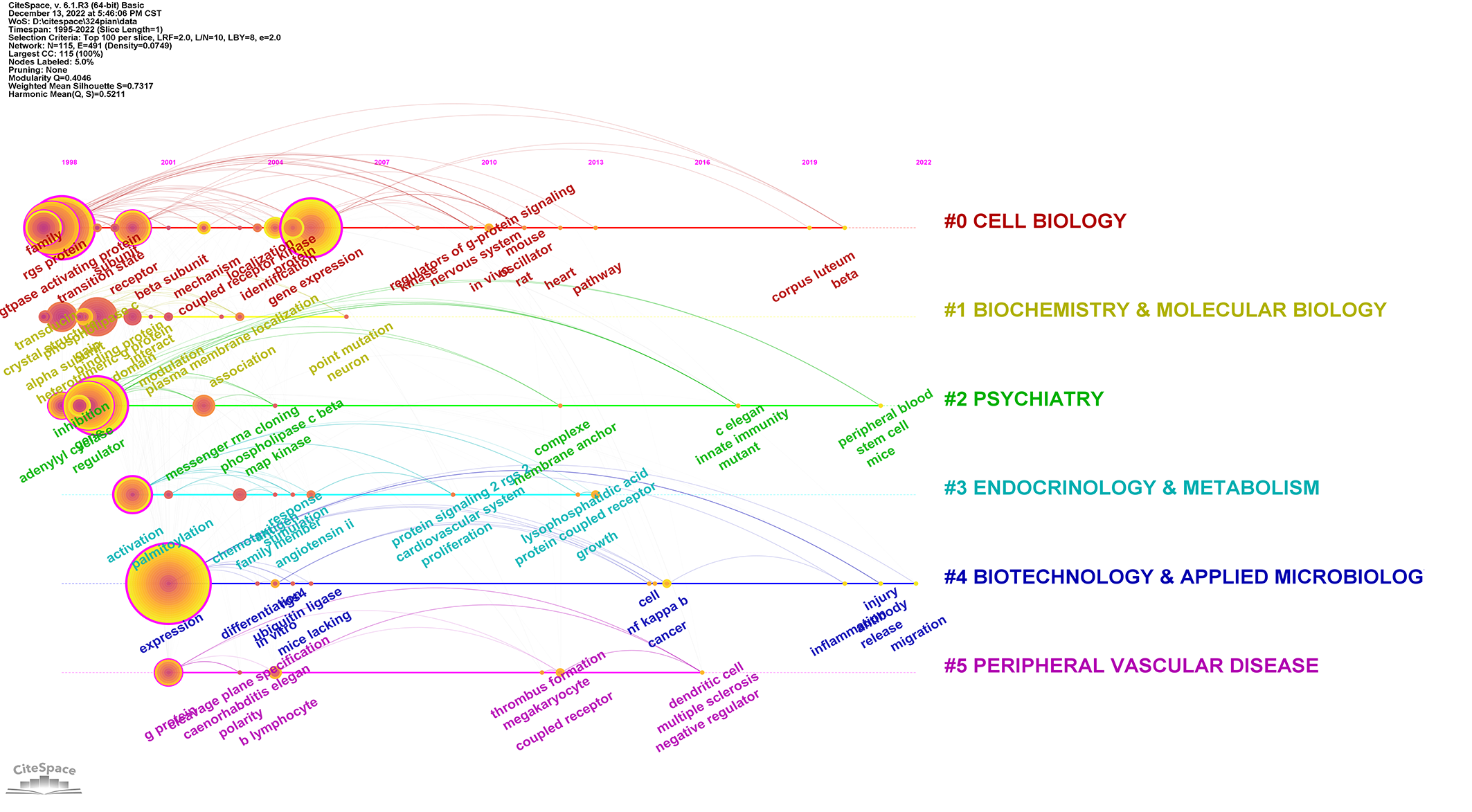

Supplement: Supplementary file 5 [file medi-103-e36981-s005.tif]

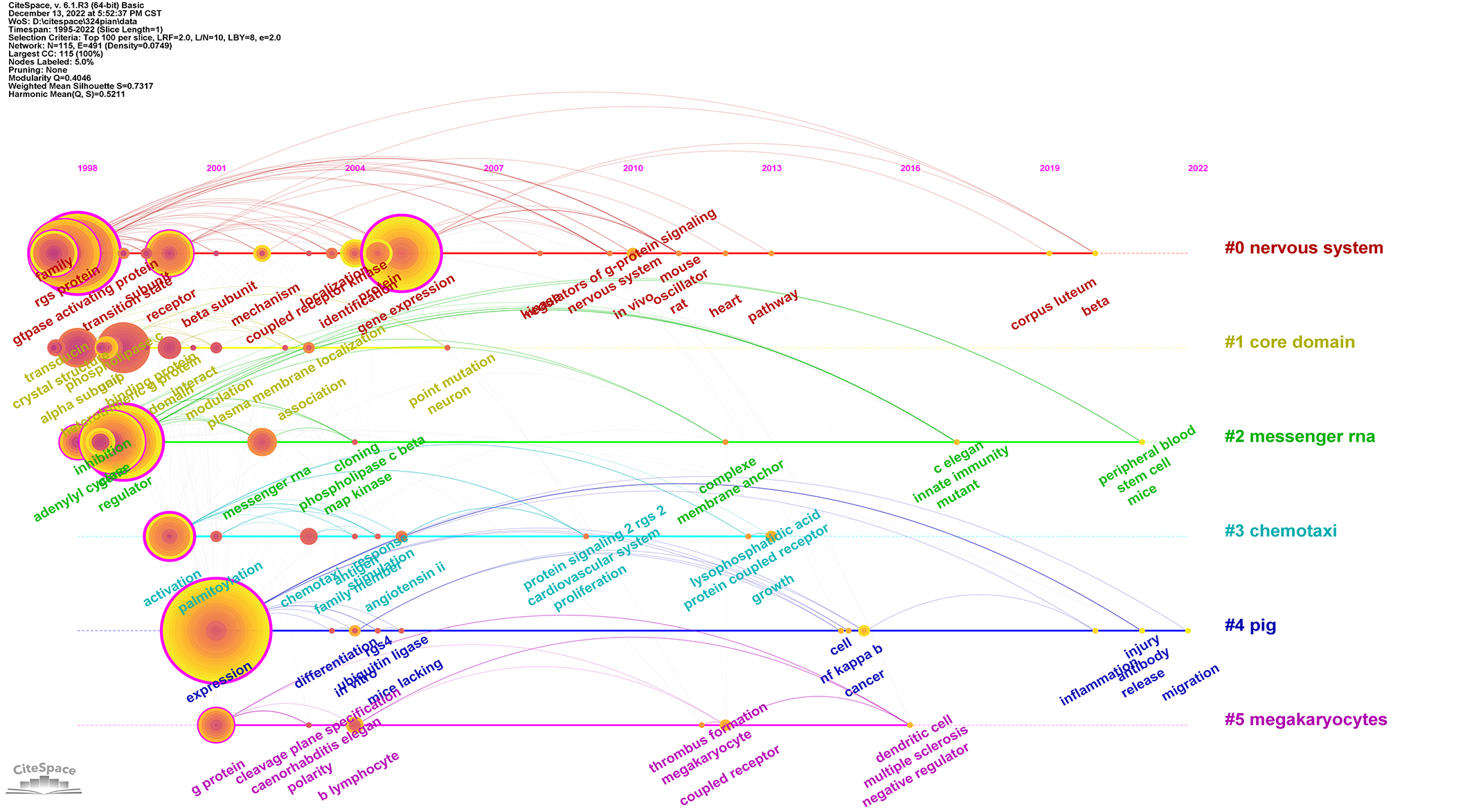

Supplement: Supplementary file 6 [file medi-103-e36981-s006.tif]

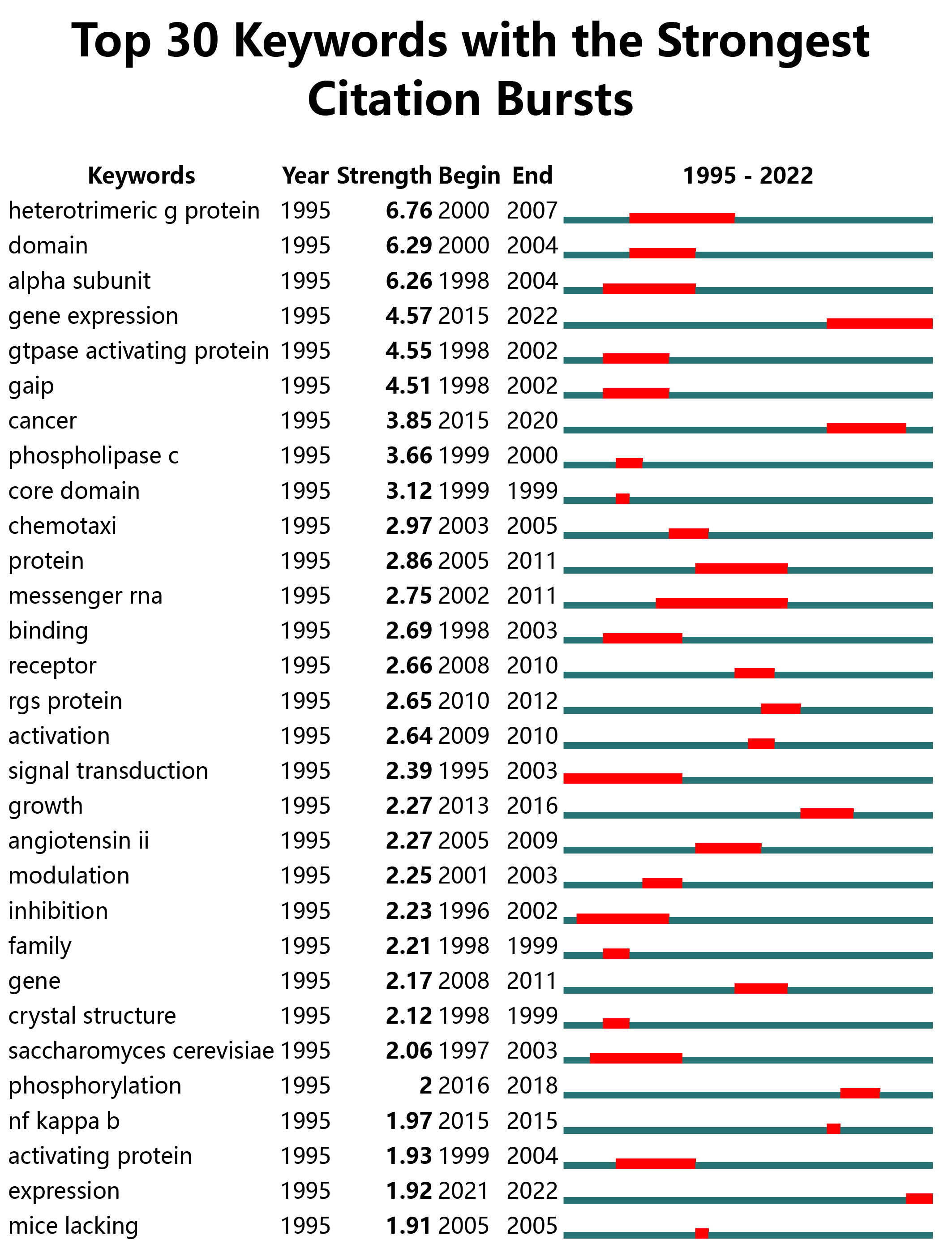

Supplement: Supplementary file 7 [file medi-103-e36981-s007.tif]

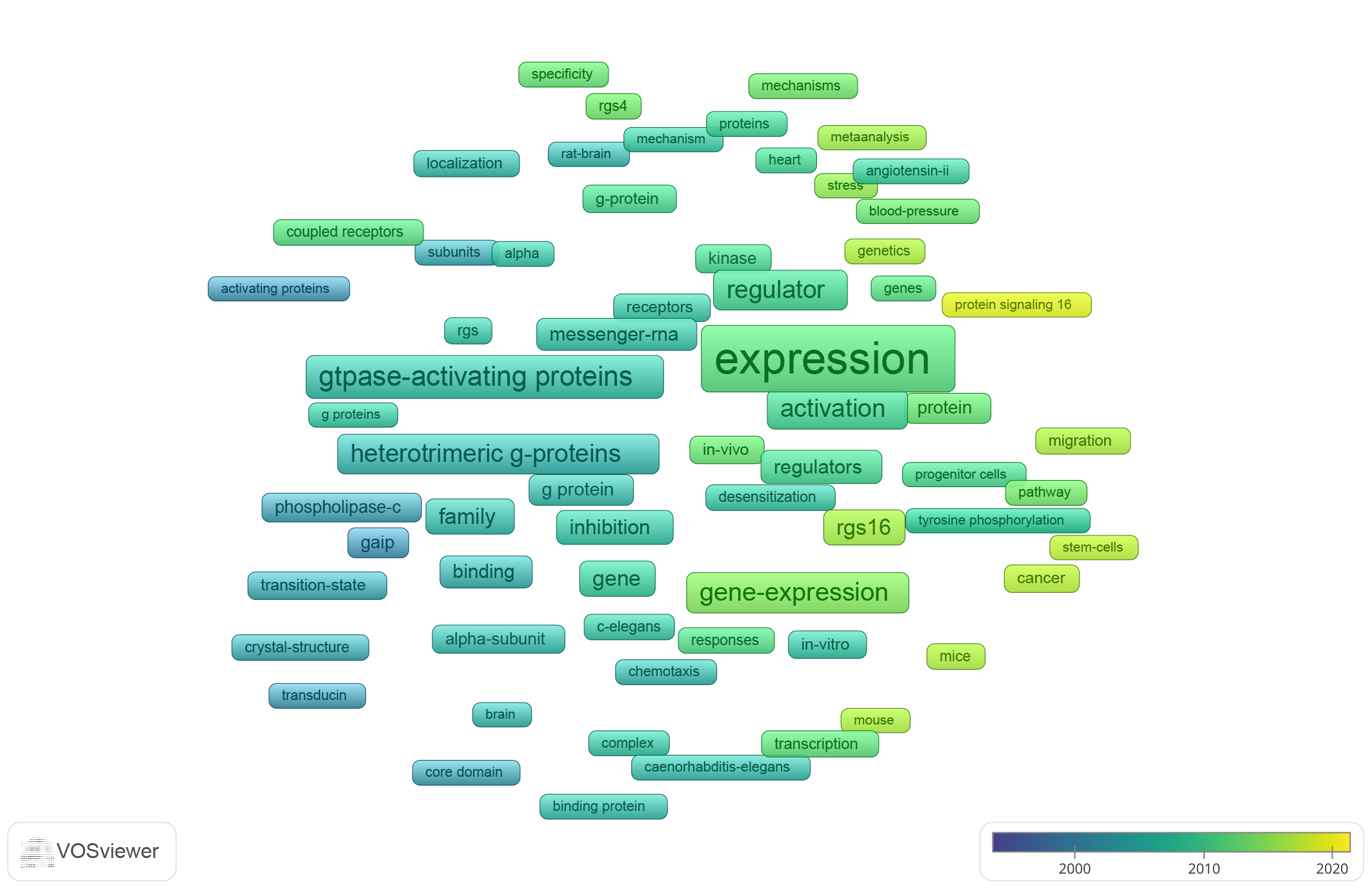

Supplement: Supplementary file 8 [file medi-103-e36981-s008.tif]

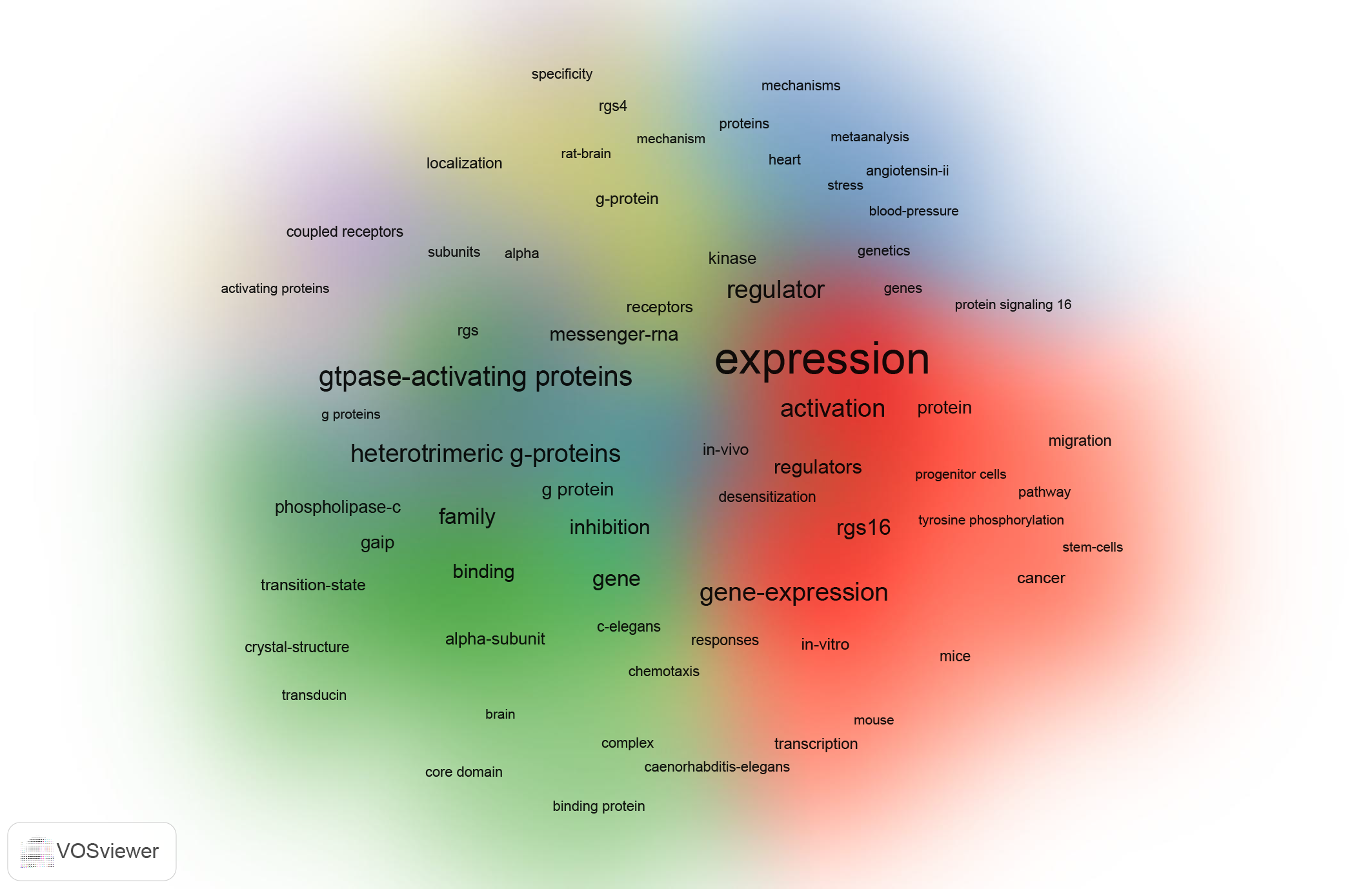

Supplement: Supplementary file 9 [file medi-103-e36981-s009.tif]

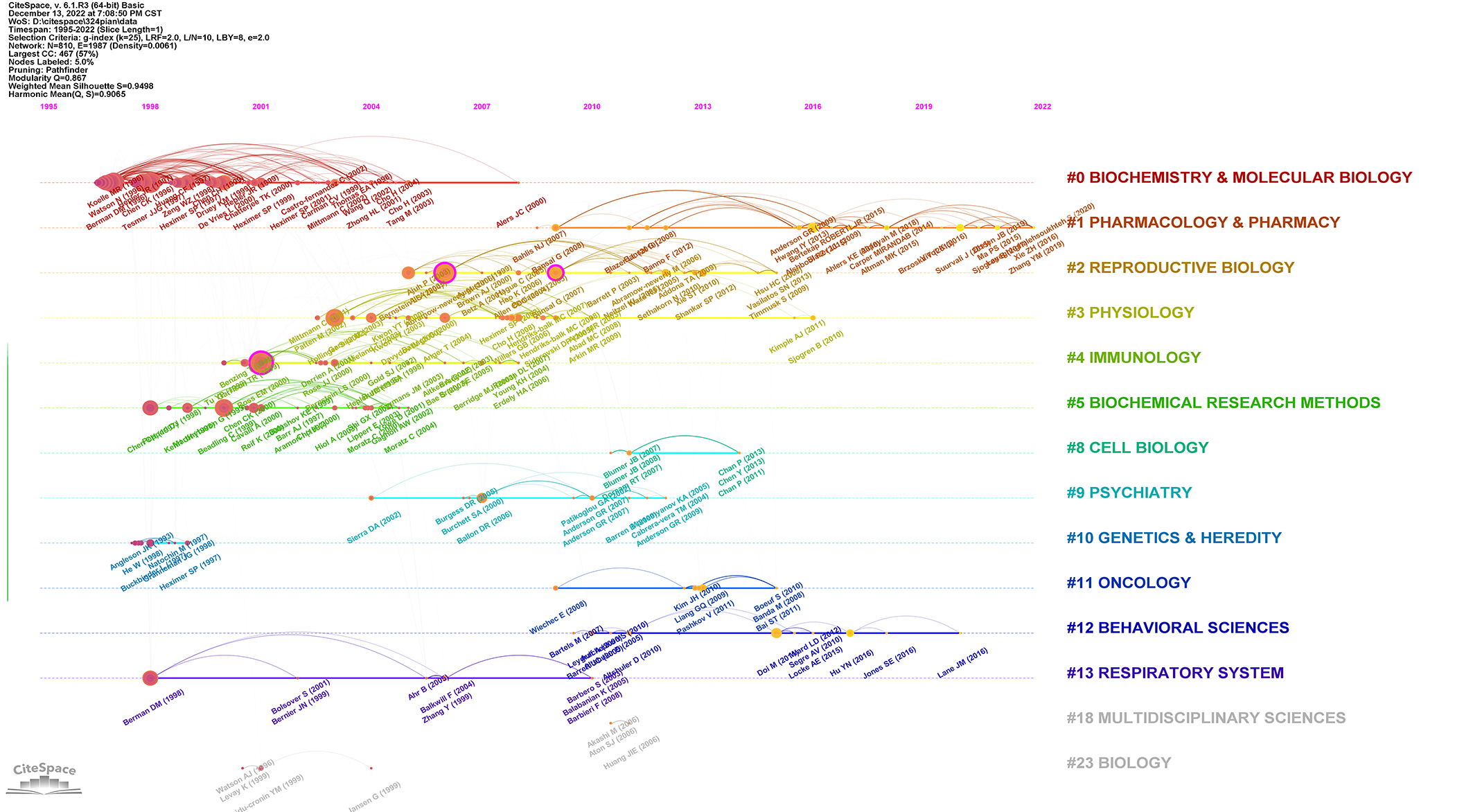

Supplement: Supplementary file 10 [file medi-103-e36981-s010.tif]

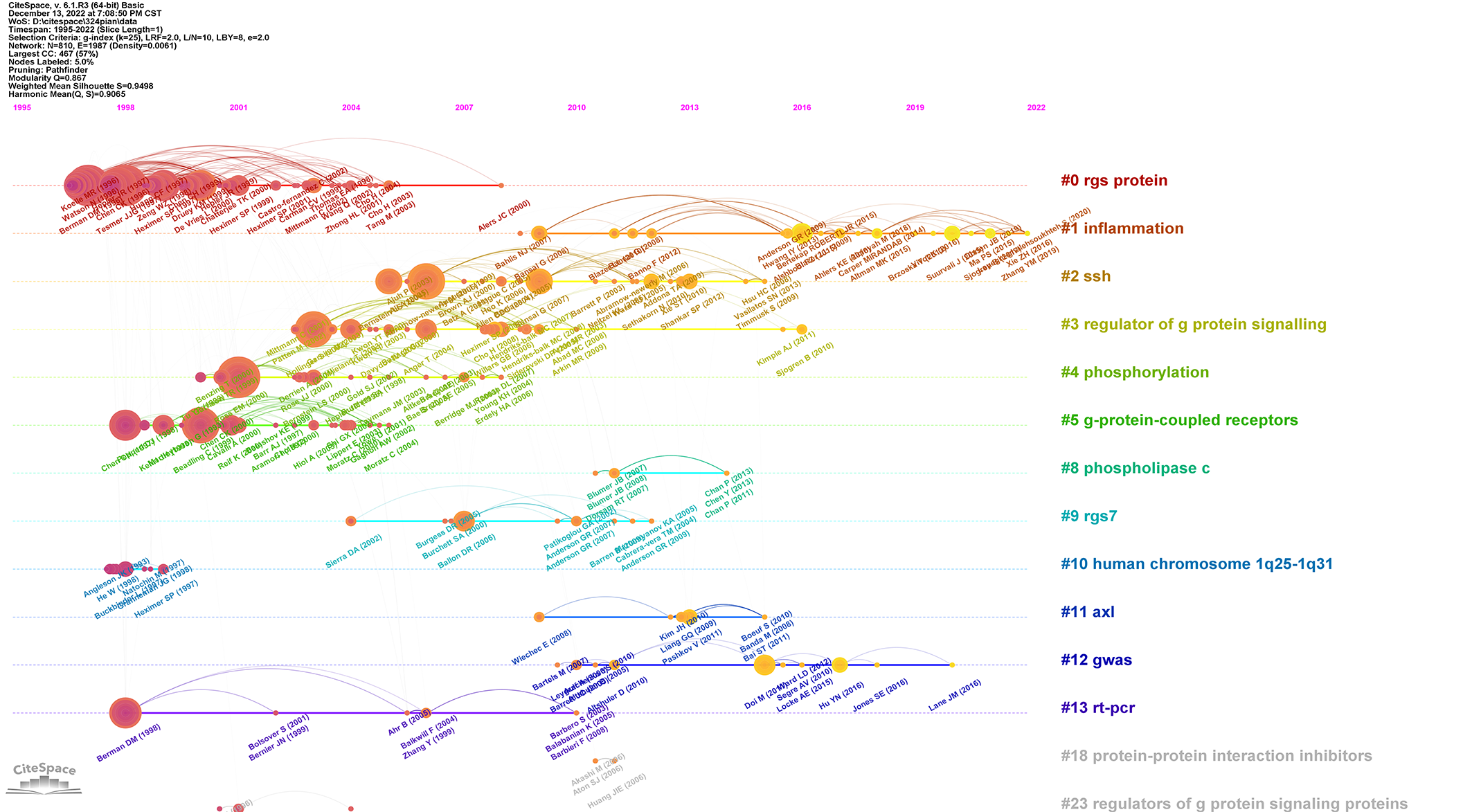

Supplement: Supplementary file 11 [file medi-103-e36981-s011.tif]

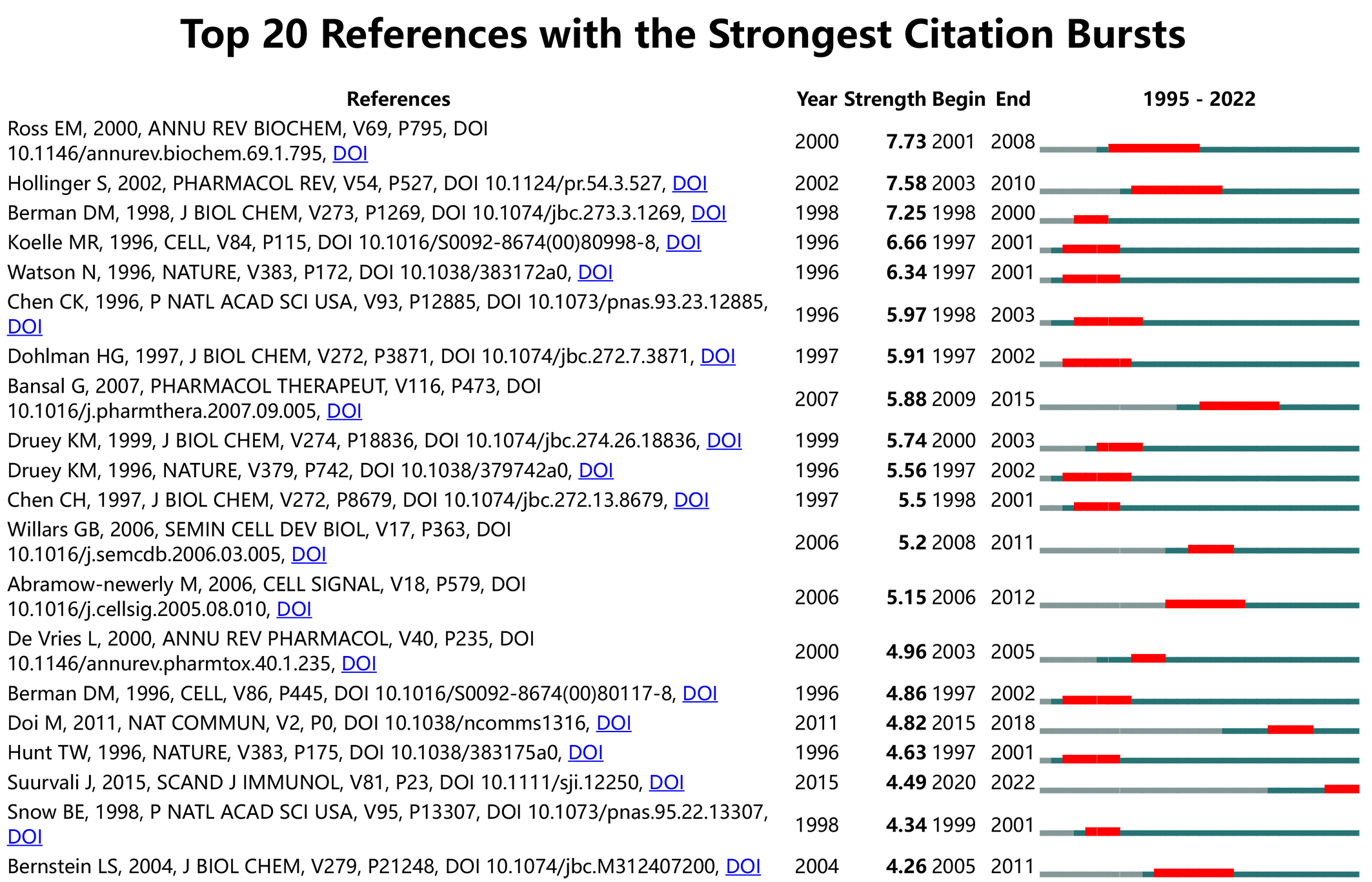

Supplement: Supplementary file 12 [file medi-103-e36981-s012.tif]

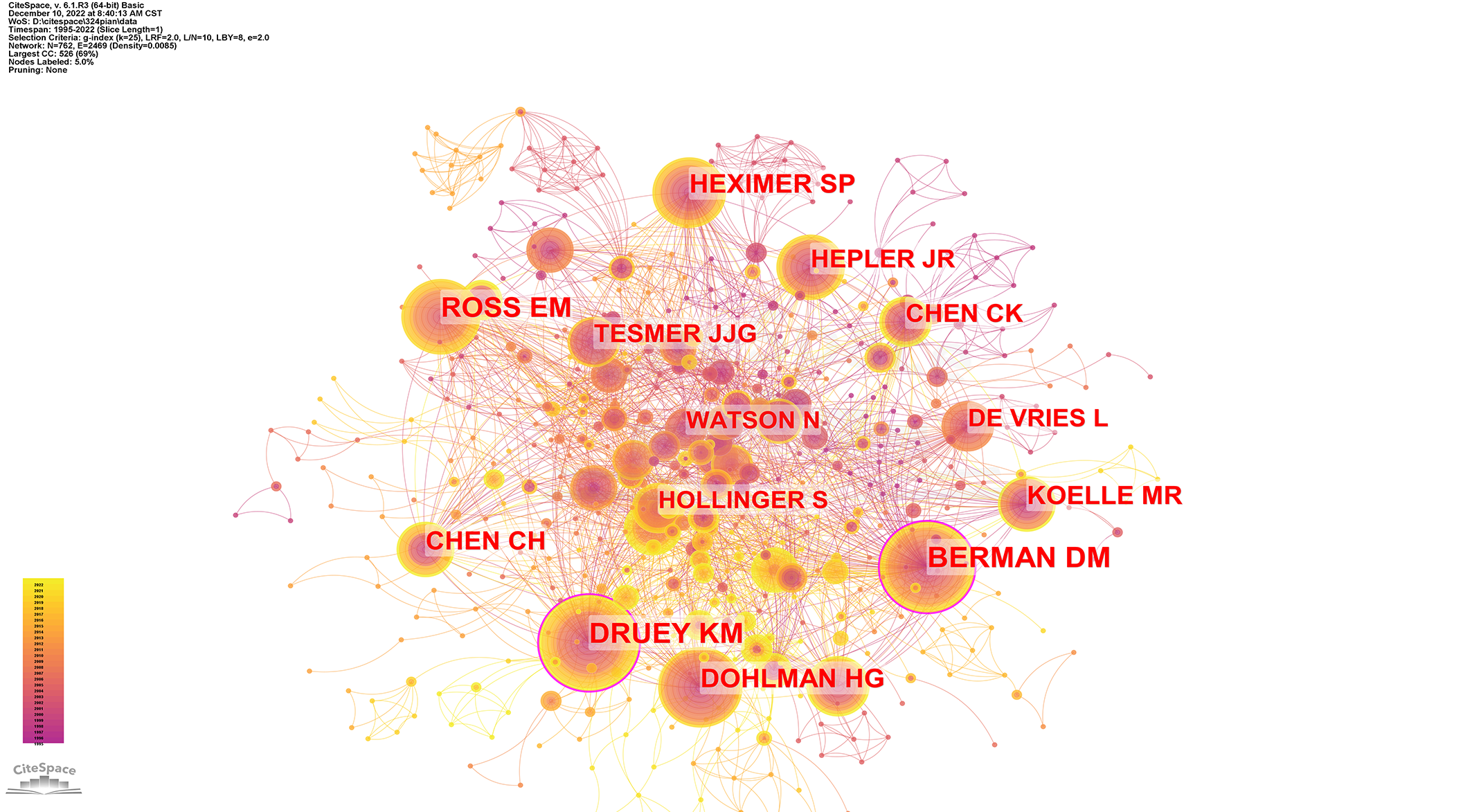

Supplement: Supplementary file 13 [file medi-103-e36981-s013.tif]

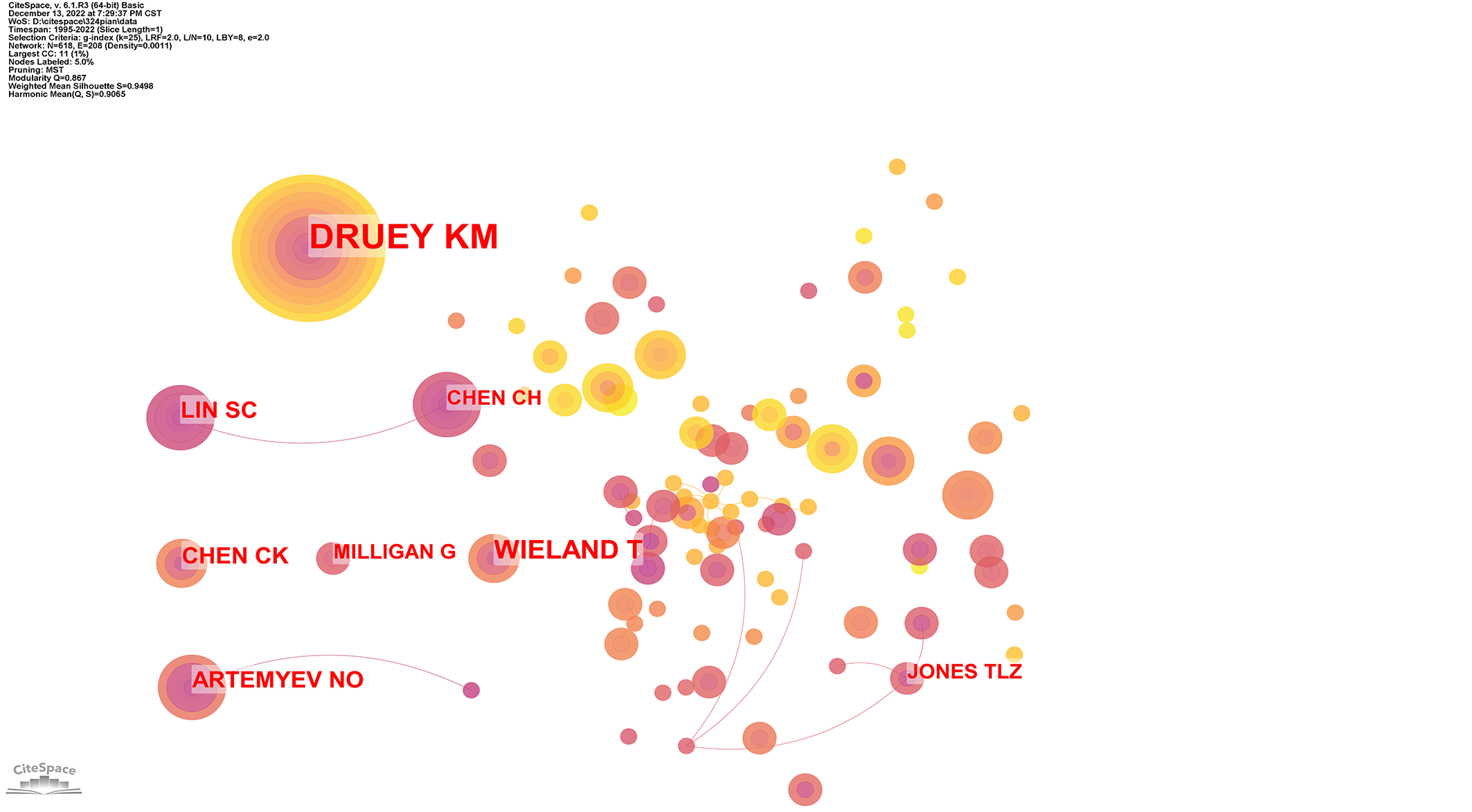

Supplement: Supplementary file 14 [file medi-103-e36981-s014.tif]

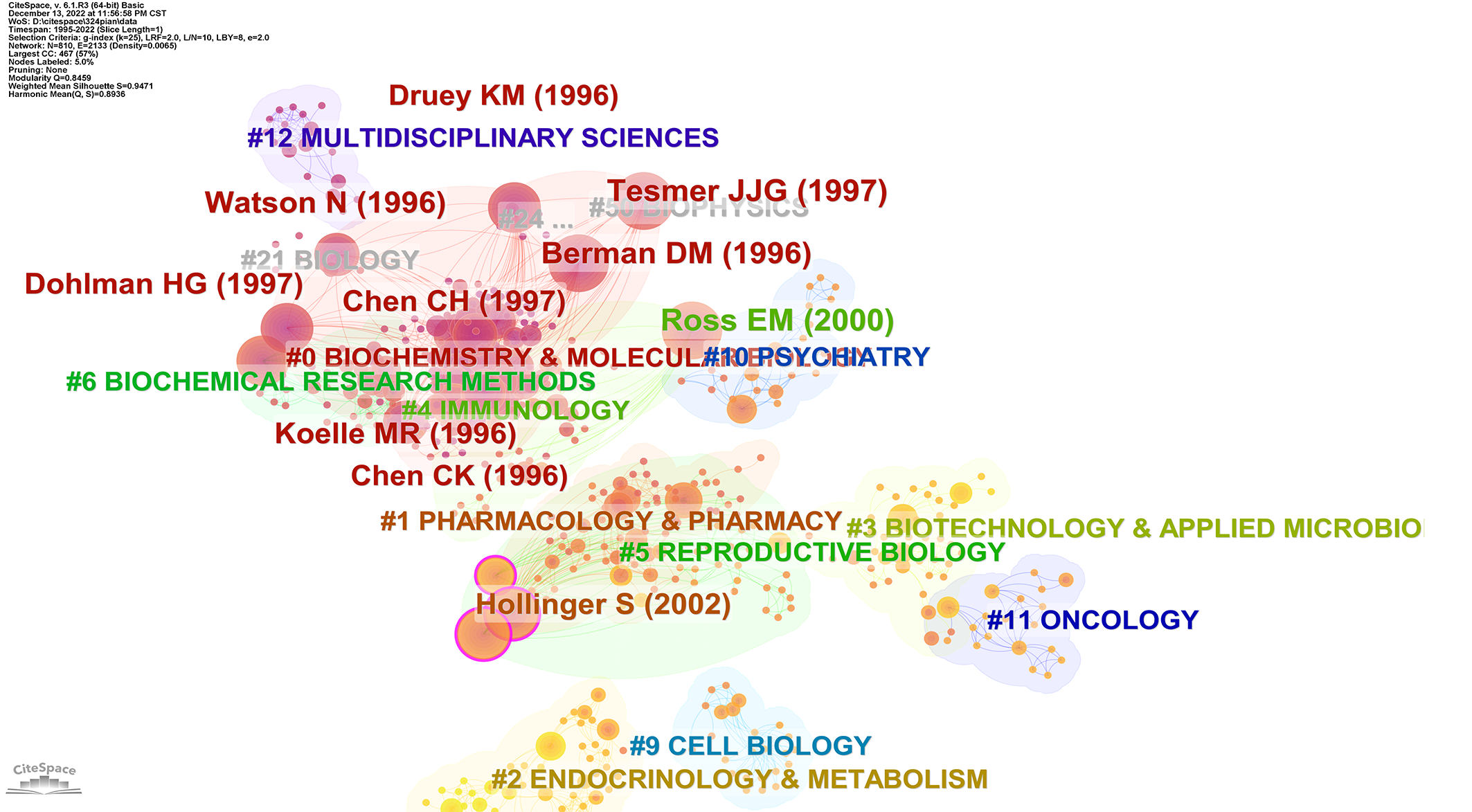

Supplement: Supplementary file 15 [file medi-103-e36981-s015.tif]

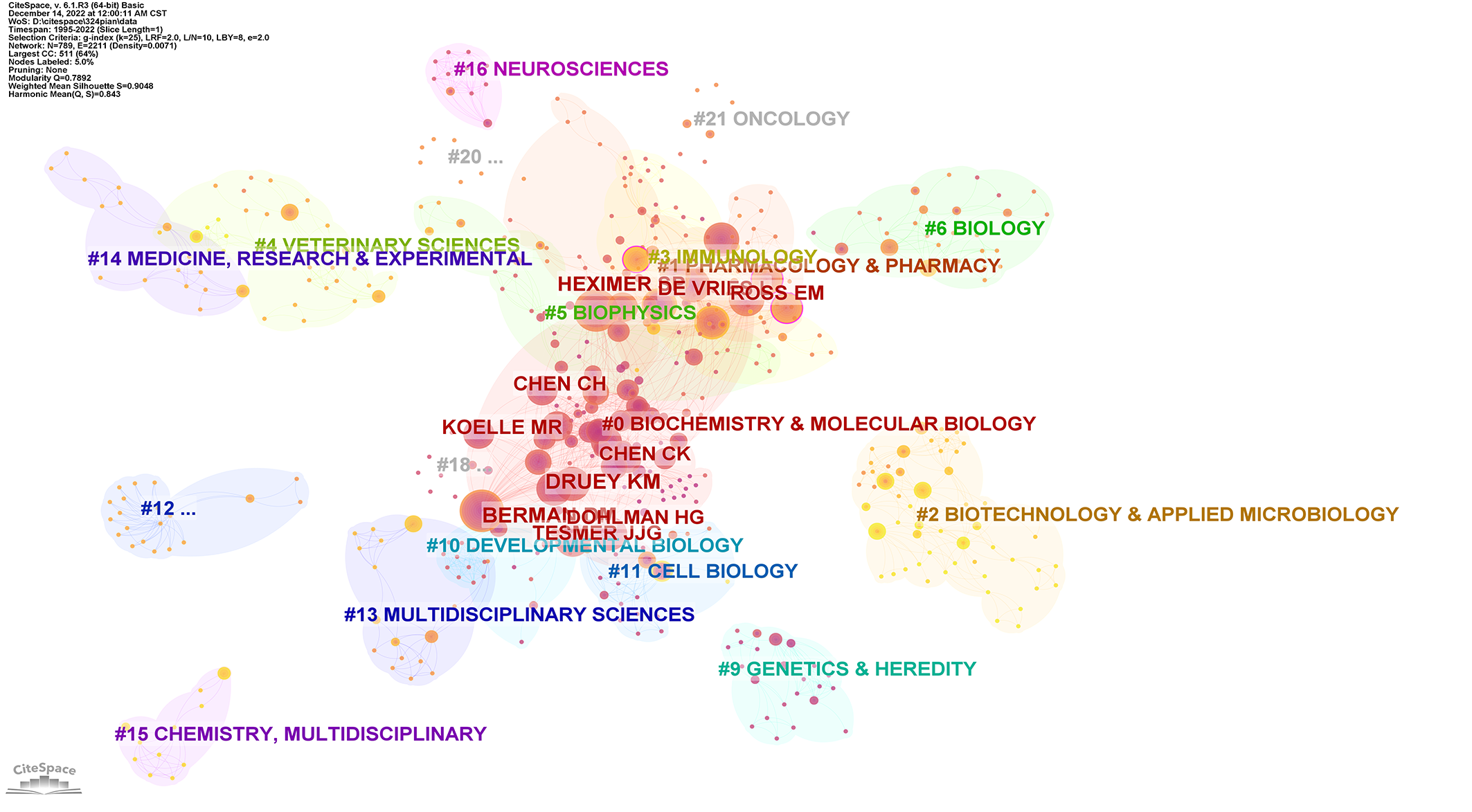

Supplement: Supplementary file 16 [file medi-103-e36981-s016.tif]

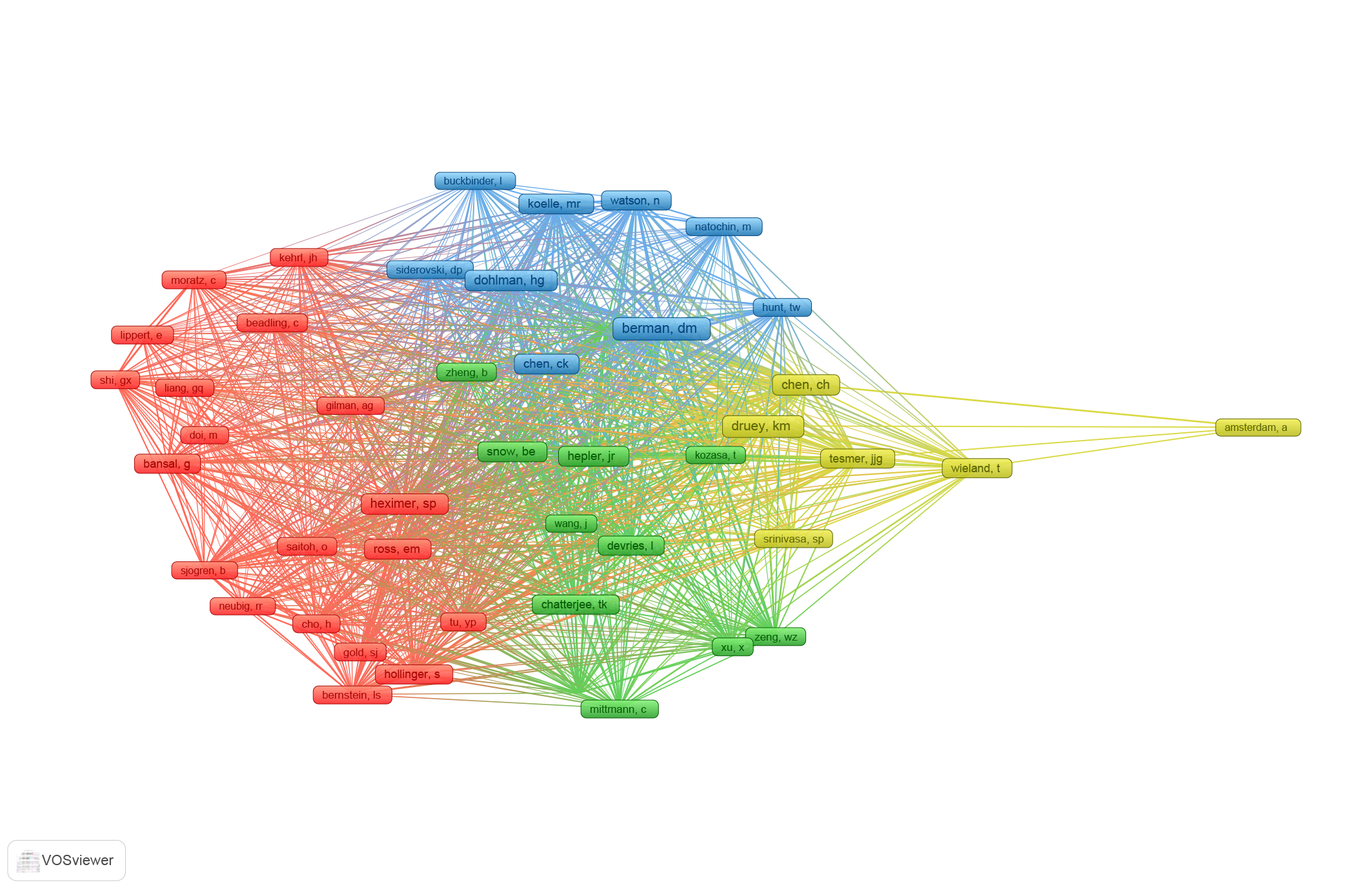

Supplement: Supplementary file 17 [file medi-103-e36981-s017.tif]

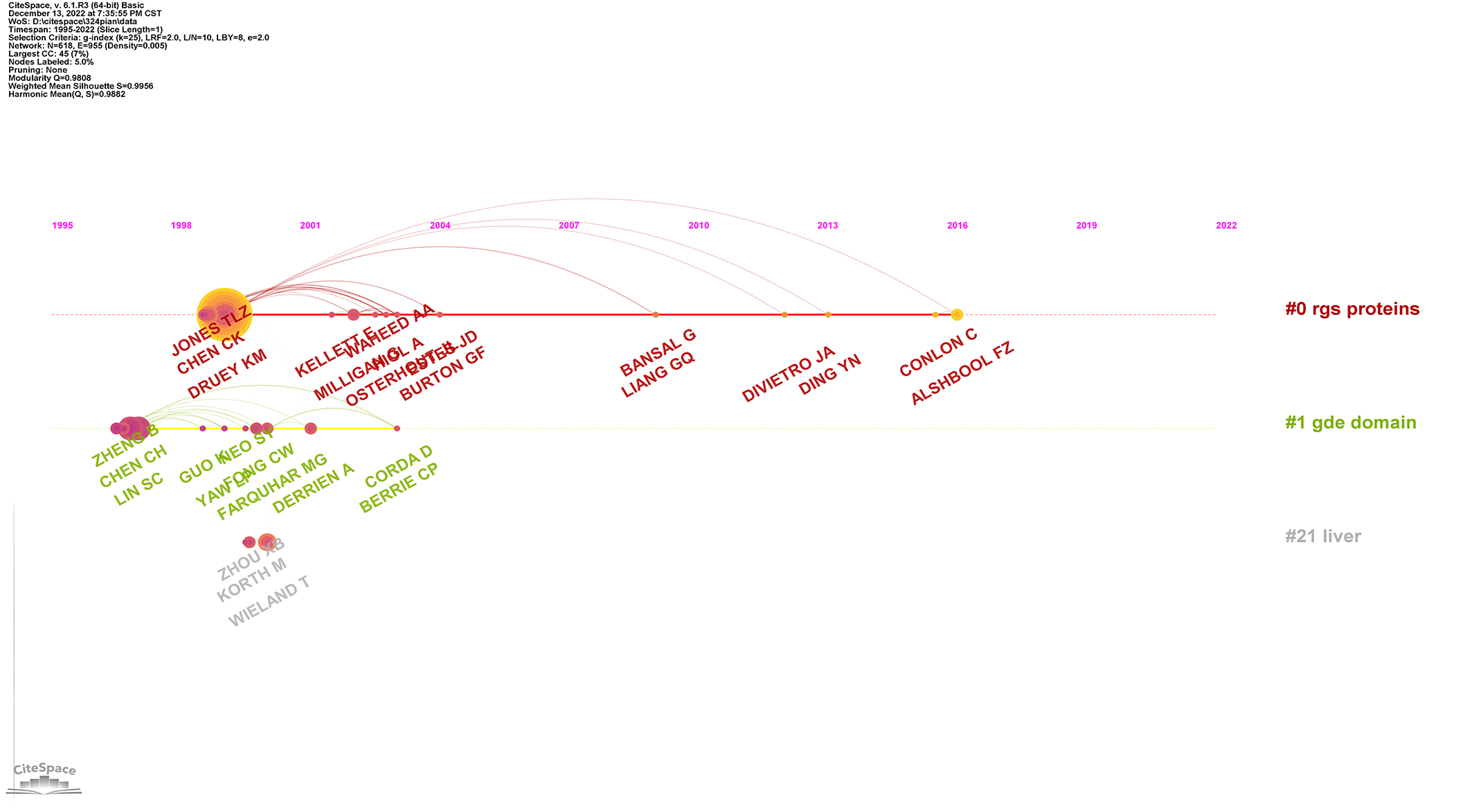

Supplement: Supplementary file 18 [file medi-103-e36981-s018.tif]

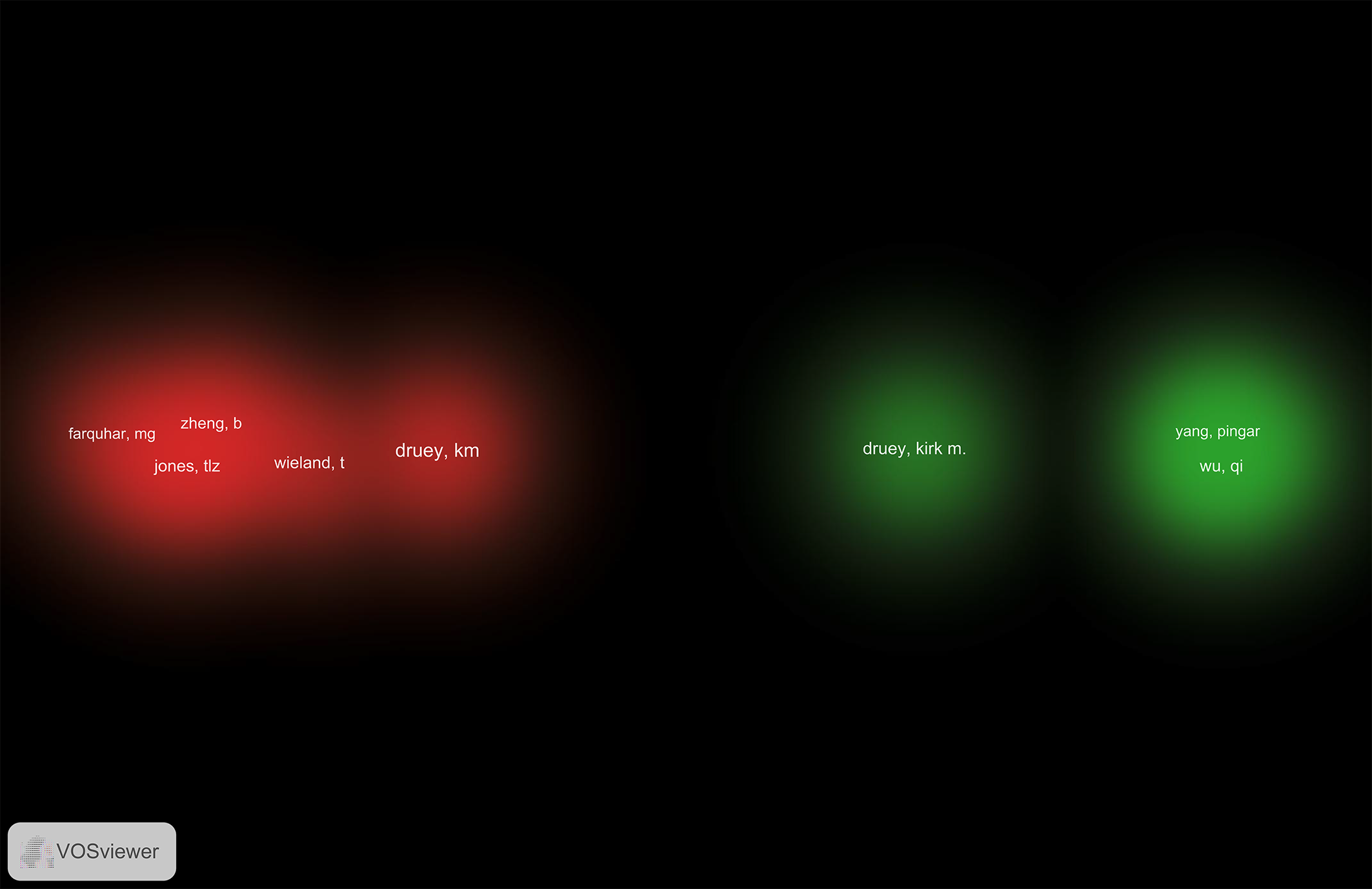

Supplement: Supplementary file 19 [file medi-103-e36981-s019.tif]

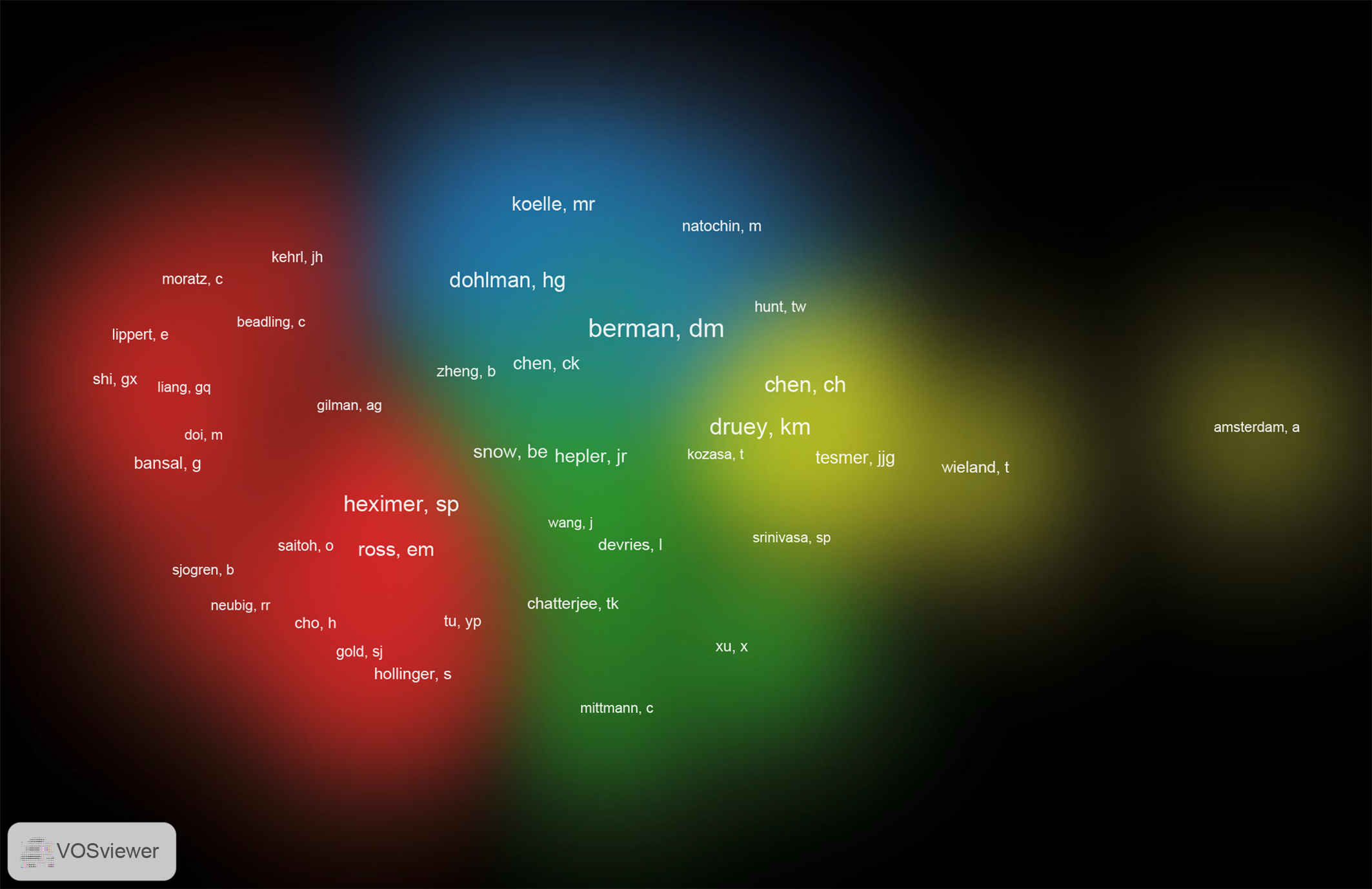

Supplement: Supplementary file 20 [file medi-103-e36981-s020.tif]
